# Unveiling the Synergy between Surface Terminations and Boron Configuration in Boron-Based Ti_3_C_2_ MXenes Electrocatalysts for Nitrogen Reduction Reaction

**DOI:** 10.1021/acscatal.4c03415

**Published:** 2024-10-03

**Authors:** Ling Meng, Francesc Viñes, Francesc Illas

**Affiliations:** Departament de Ciència de Materials i Química Física & Institut de Química Teòrica i Computacional (IQTCUB), Universitat de Barcelona, c/Martí i Franquès 1-11, 08028 Barcelona, Spain

**Keywords:** Ti_3_C_2_T_*x*_, B-doped MXene, NRR, density functional theory, surface termination

## Abstract

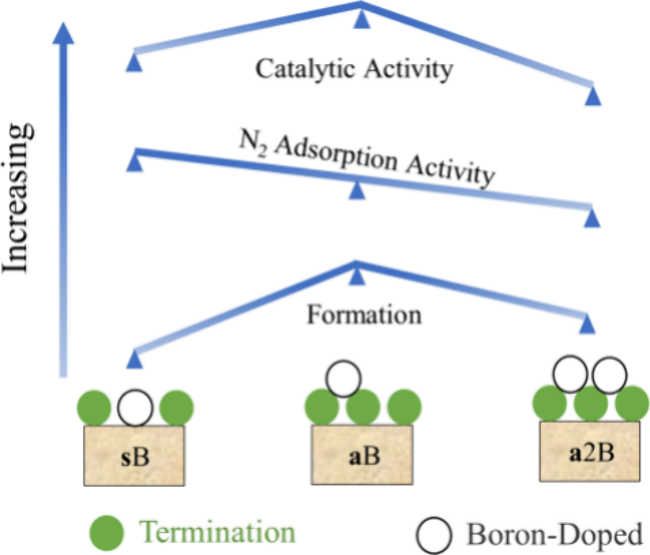

The performance of B-containing Ti_3_C_2_ MXenes as catalysts for the nitrogen reduction reaction (NRR) is scrutinized using density functional theory methods on realistic models and accounting for working conditions. The present models include substituted and adsorbed boron along with various mixed surface terminations, primarily comprising −O and −OH groups, while considering the competitive hydrogen evolution reaction (HER) as well. The results highlight that substituted and low-coordinate adsorbed boron atoms exhibit a very high N_2_ adsorption capability. For NRR, adsorbed B atoms yield lower limiting potentials, especially for surfaces with mixed −O/–OH surface groups, where the latter participate in the reaction lowering the hydrogenation reaction energy costs. The NRR does also benefit of having B adsorbed on the surface which on moderate −OH terminated model displays the lowest limiting potential of −0.83 V, competitive to reference Ru and to HER. The insights derived from this comprehensive study provide guidance in formulating effective MXene-based electrocatalysts for NRR.

## 1 Introduction

Gas phase nitrogen (N_2_), having a strong triple bond, is one of the most abundant nitrogen containing compounds on Earth, and it is heavily utilized in the chemical industry for the conventional Haber-Bosch process to synthesize ammonia (NH_3_). Ammonia can then become oxidized to nitric acid, which constitutes a key component in global fertilizer production.^1,2^ The Haber–Bosch industrial process requires not only the presence of a catalyst but also high temperature (above 350 °C) and high pressure (above 150 atm) conditions, implying a huge amount of energy consumption with concomitant serious carbon dioxide (CO_2_) emissions.^3,4^ Therefore, in the pursuit of long-term sustainability, alternative NH_3_ synthesis technologies working at softer conditions are being actively explored.^5,6^

Inspired by natural biological N_2_ fixation,^7^ finding ammonia synthesis procedures working at mild conditions (room temperature and atmospheric pressure) constitutes a challenge and an exciting alternative.^8,9^ Particularly, the electrocatalytic nitrogen reduction reaction (NRR) has garnered significant attention as an appealing carbon-neutral methods,^10,11^ especially when electricity can be obtained from renewable sources.^12^ Ammonia electrosynthesis typically involves a coordinated transfer of protons and electrons, with protons sourced from aqueous medium and electrons from renewable electrical resources,^12^ thus avoiding CO_2_ emissions as well.^13^ However, this technological appealing process is hampered by the lack of efficient electrocatalysts fulfilling the strict limitations of NRR.^14^ Therefore, the development of efficient and highly selective NRR electrocatalysts holds significant economic importance and urgency.

In recent years, a new class of two-dimensional (2D) materials known as MXenes^15−17^ have emerged as a frontier with ever-growing technological applications.^18^ MXenes are new types of 2D transition metal carbides, nitrides, and carbonitrides, with general M_*n*+1_X_*n*_T_*x*_ (*n* = 1–3) formula, consisting of early transition metals (M), carbon, and/or nitrogen (X), and typically with terminal functional groups, T_*x*_, usually −O, −H, −OH, −F,^16,19,20^ or a combination of them.^21,22^ Recently, there has been considerable interest in using MXenes as a catalyst for the NRR.^23−26^ For instance, it has been reported that the M_2_C(0001) surface of several MXenes readily adsorbs and dissociates N_2_.^8,27^ Azofra *et al*.^28^ conducted a computational analysis of the NRR on M_3_C_2_ MXenes aimed at evaluating their capability in capturing, activating, and electrochemically transforming N_2_ into NH_3_. These authors identified V_3_C_2_ and Nb_3_C_2_ as the most promising candidates with associated overpotentials of 0.64 and 0.90 V, respectively.

However, one must advert that these predictions were obtained from models involving the clean, bare, surface of these MXene materials, which do not appropriately represent the systems in which the reactions are taking place which involve an electrochemical environment.^21,24,29^ Explicitly accounting for a given termination in the M_2_XT_*x*_ of T_*x*_ = −H, −O, −N, Johnson *et al*.^24^ reported a strong influence of the termination on the predicted free energy profiles. However, mixed terminations need to be considered in real-world scenarios.^21,22,30,31^ In fact, the type and quantity of surface terminations of MXene are highly sensitive to synthesis conditions such as etching time, temperature, or etchant concentration.^32,33^ For instance, typically, hydrofluoric acid (HF) is used as the etchant; however, higher concentrations of HF tend to result in more −F terminations, while lower concentrations lead to the prevalence of −O species.^34^ Furthermore, successful synthesis protocols with F-free and gaining T_*x*_-free MXenes have also been reported.^35−38^

There is experimental evidence that surface engineering of MXenes, particularly the types and concentrations of surface terminations, is crucial in facilitating electron transfer, surface adsorption, and the activation of N_2_. For instance, Xia *et al*.^39^ found that NH_3_ production is enhanced with the increase of −OH groups at the Ti_3_C_2_ MXene surface; furthermore, Ding *et al*.^40^ reported experimentally that Ti_3_C_2_T_*x*_ MXene with medium density F-functionalized terminations could enhance the adsorption and activation of N_2_. Clearly, the aforementioned theoretical studies not considering the role of terminations are not realistic, and more in-depth studies are needed to fill this knowledge gap and better understand the intricate details of MXene surface engineering in the context of NRR.

The present work focus is on Ti_3_C_2_, the first MXene ever synthesized.^15^ Previous research indicated that the uneven distribution of surface functional groups on MXenes can result in the introduction of numerous oxygen vacancies on the MXene surface that can be occupied by other elements.^41,42^ The case of boron atoms is particularly attractive since there is evidence that it exhibits a great potential for N_2_ fixation.^43,44^ In fact, several B-based NRR electrocatalysts, based on g-C_3_N_4_, graphene, and 2D boron sheets, have been reported,^45−47^ and some studies have been conducted on B-based MXenes for NRR.^48−50^ However, these previous studies used oversimplified models, considering scenarios where B substitutes the −O surface termination only but where its existence, vicinity of terminations, and B quantity were not adequately represented, which calls for more elaborated studies.

**Figure 1:**
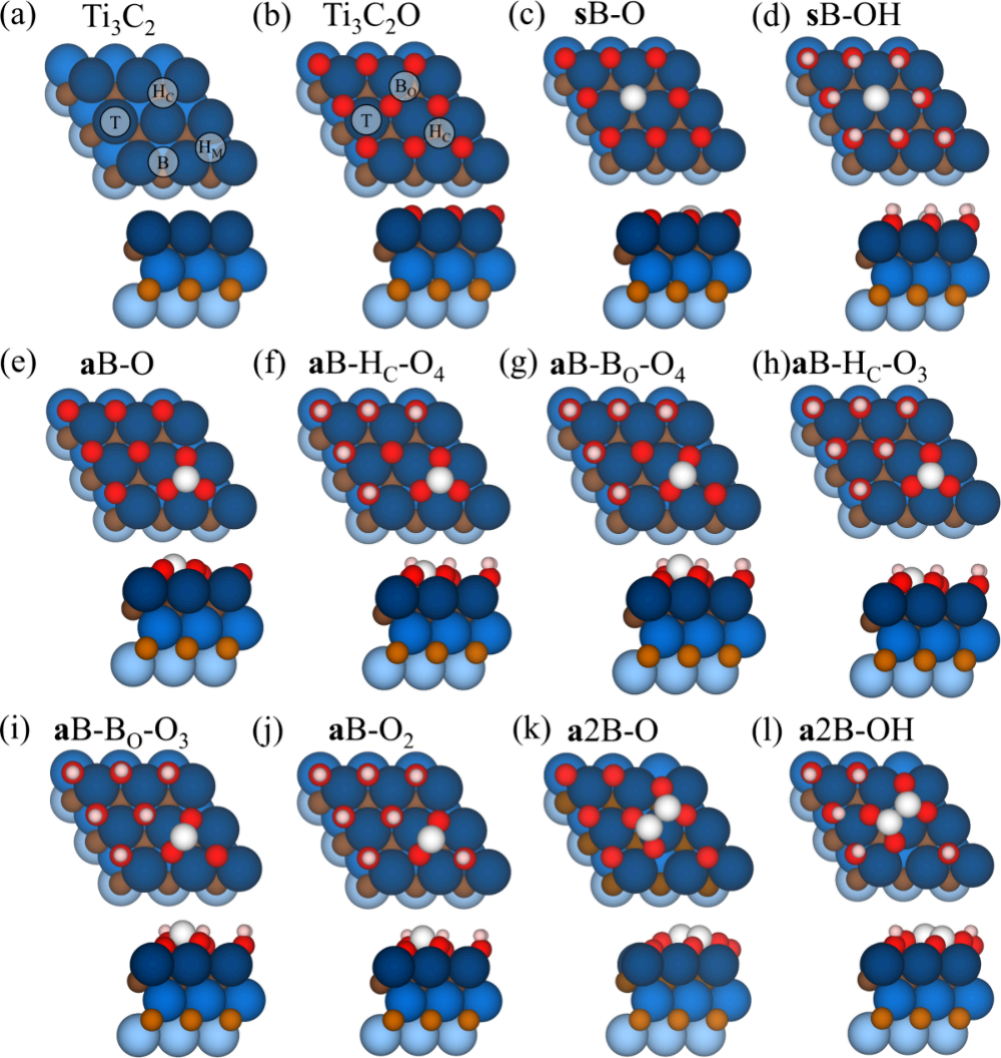
Top (upper images) and side (lower images) views of the ten models derived from the employed *p*(3×3) MXene supercells, including (a) pristine Ti_3_C_2_ (0001) with the top (T), bridge (B), hollow carbon (H_C_), and hollow metal (H_M_) four high-symmetry sites tagged; (b) Ti_3_C_2_O (0001) with T, H_C_, and O-bridge (B_O_) three high-symmetry sites tagged; (c) **s**B@Ti_3_C_2_O_8/9_ (**s**B–O); (d) **s**B@Ti_3_C_2_OH_8/9_ (**s**B–OH); (e) **a**B@Ti_3_C_2_O with B on H_C_ (**a**B–O); (f) **a**B@Ti_3_C_2_O_4/9_OH_5/9_ with B on H_C_ (**a**B–H_C_-O_4_); (g) **a**B@Ti_3_C_2_O_4/9_OH_5/9_ with B on B_O_ (**a**B–B_O_-O_4_); (h) **a**B@Ti_3_C_2_O_1/3_OH_2/3_ with B on H_C_ (**a**B–H_C_-O_3_); (i) **a**B@Ti_3_C_2_O_1/3_OH_2/3_ with B on B_O_ (**a**B–B_O_-O_3_); (j) **a**B@Ti_3_C_2_O_2/9_OH_7/9_ (**a**B–O_2_); (k) **a**2B@Ti_3_C_2_O (**a**2B–O), and (l) **a**2B@Ti_3_C_2_O_4/9_OH_5/9_ (**a**2B–O_4_). H and O atoms in the termination groups are represented by light pink and red spheres, respectively, while B atoms are represented by white spheres. Ti and C atoms are shown as blue and brown spheres with different levels of shading depending on their stacking position, with darker versions being closer to the shown surface.

There is, regretfully, no current research specifically demonstrating the potential adsorption of boron atoms on the terminal groups of MXenes. Therefore, following earlier studies of single-boron MXene NRR catalysts, we address the present research using ten unique models based on Ti_3_C_2_T_*x*_, specifically encompassing different types of situations for B atoms, as shown in Figure 1, with additional details given below and also in Table S1 of the Supporting Information (SI). By using these models, one can obtain detailed insights into the NRR process under realistic working conditions. In particular, the present results highlight (*i*) that the stability of the catalysts is influenced by the arrangement of B (substituted/adsorbed), which may result in completely opposite stability behavior at the same termination; (*ii*) the N_2_ activation capacity of the selected models considering the working temperature, *T*, and N_2_ partial pressure, *p*_N_2__, as well as the influence of the coordination number and surface groups on them; (*iii*) the effects of −O and −OH groups on NRR under acidic conditions, assuming that MXene is synthesized through a F-free method; in addition, (*iv*) we propose a new NRR mechanism that involves different terminations and B-scenarios, where, (*v*) contrary to previous works where only substituted B were considered, situations with adsorbed B are not only more stable but also exhibit enhanced performance in NRR; and finally, (*vi*) the situations with moderate −OH coverage exhibit the most excellent NRR activity and selectivity, with feasibility points in their experimental synthesis and use.

## 2 Computational Details

### 2.1 Models and Methods

The Vienna *Ab initio* Simulation Package (VASP)^51^ was used to carry out all the needed periodic density functional theory (DFT) calculations. The generalized gradient approximation (GGA) Perdew–Burke–Ernzerhof (PBE)^52^ exchange–correlation functional was employed and found to describe the electronic structure and related properties of MXenes with sufficient accuracy.^53−55^ Furthermore, the D3 approach proposed by Grimme *et al*.^56^ was used to account for interactions involving dispersion. The valence electron density was expanded using a plane wave basis set, with a cutoff kinetic energy of 415 eV, to ensure that the total energies obtained were converged below the chemical accuracy of 1 kcal mol^–1^ —*ca.* 0.04 eV,^57^ whereas the projector augmented wave (PAW) method,^58^ as implemented by Kresse and Joubert,^59^ was employed to describe the effect of core electrons on the valence electron density.

Utilizing a *p*(3×3) supercell, see Figure 1, a F-free experimental protocol is assumed to initially lead to fully −O or −OH covered Ti_3_C_2_. Subsequently, considering the superior stability of −OH termination according to Pourbaix diagrams and the abovementioned influence of the vicinity of B, the mixed −O and −OH cases with ^2^/_9_ vs ^7^/_9_ of a monolayer (ML), ^1^/_3_ vs ^2^/_3_ ML, and ^4^/_9_ vs ^5^/_9_ ML were also explored, respectively. Note that here a full ML is defined as having one surface moiety per surface metal atom. In addition, a 20 Å vacuum was added to the periodically repeated slabs to ensure their appropriate isolation. Numerical integration in the Brillouin zone was carried out on an optimized 5×5×1 **k**-point **Γ**-centered Monkhorst–Pack grid,^60^ providing numerical convergence within 0.05 eV, very close to the accepted chemical accuracy value of *ca*. 0.04 eV. The energy of isolated atoms and molecules at the equilibrium geometry in vacuum was determined by carrying calculations in a broken symmetry large cell of 10×10×10 Å^3^ dimensions to ensure due orbital occupancy, carried out spin-polarized for systems with unpaired electrons, and performed at **Γ**-point only.

During the structural optimizations, the convergence of the electronic self-consistent field steps was controlled using a criterion of 10^–5^ eV, and the relaxation of atomic positions was performed until the forces exerted on the atoms were below 0.01 eV Å^–1^. It should be noted that unless specified otherwise, all calculations were conducted taking spin polarization into account. The vibrational frequencies of the stationary points related to surface species were determined by constructing and diagonalizing the corresponding block of the Hessian matrix using finite differences of analytical gradients with steps of 0.03 Å in length as done in previous works.^61,62^ Thus, only degrees of freedom involving the adsorbed species and terminations were accounted, while the substrate atoms were kept fixed, assuming a decoupling of the surface species from the material phonons.

On the above models, adsorbed (**a**) and substituted (**s**) B situations with single B or B pairs were examined, also considering two different B coordination types on H_C_ or B_O_ sites, plus different surface situations for the terminating groups. The complete set of models used and the corresponding notation are displayed in Figure 1. To better follow the employed notation, let us take the **s**B@Ti_3_C_2_O_8/9_ and **a**2B@Ti_3_C_2_O_4/9_OH_5/9_ cases as examples. One can readily see that a single B substitutes one surface −O group on Ti_3_C_2_O_8/9_, whereas two B are adsorbed on the Ti_3_C_2_O_4/9_OH_5/9_ model. For each model, the symbols in parentheses in Figure 1 are used as a shortcut for notation simplification.

### 2.2 NRR Mechanism

The initial, *sine qua non*, step in the NRR is gas phase, N_2_^(g)^, adsorption onto a catalyst-free surface site, *, as;
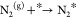
1

The subsequent reaction mechanism involves a series of concerted proton–electron transfer (CPET) steps, where protons (H^+^) and electrons (e^–^) are added together, but in a sequential manner up to ammonia (NH_3_) product.^63^ Thus;
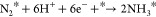
2

Next, it is highly likely that NH_3_* will dissolve in water, resulting in the formation of NH_4_OH_(l)_,^64,65^ as;
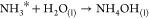
3and, because of this, NH_3_ desorption is not considered a determining step.

For convenience, it is often assumed that the reduction of precursor adsorbed species, A*, occurs in a single elementary reaction step,^66,67^ in which H^+^ and e^–^ are directly involved in the production of AH* as;
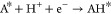
4

In addition, we also considered the potential contribution of −OH termination as a source of hydrogen. Thus, A* also could undergo hydrogenation through the −OH group, as;
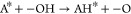
5leaving the −O moiety behind in the chemical step. Subsequently, a CPET electrochemical step can regenerate the −OH group, as;
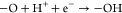
6

The present study explores various pathways to enhance the comprehension and engagement of surface terminations during the NRR in Ti_3_C_2_ MXene and its subsequent mechanism.

### 2.3 Stability of B-Based Models

To gain information about the experimental synthetic feasibility, the thermodynamic stability of the B-based MXene models was assessed. Typically, the structural stability of these concept electrocatalysts can be evaluated using parameters such as the B mean adsorption energy, *E*_ads_, estimated as
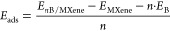
7where *E*_*n*B/MXene_, *E*_MXene_, and *E*_B_ are the total energies of MXene with and without *n* B atoms and the isolated B atom energy. As commonly done in the literature, one can compare *E*_ads_ to the computed boron bulk cohesive energy, *E*_coh_,^68−70^ in order to assess the thermodynamic stability —sometimes referred as *E*_diff_ = *E*_ads_ – *E*_coh_— so that an adsorption stronger than cohesive energy would energetically drive the presence of isolated B atoms. The B bulk cohesive energy was computed here optimizing bulk boron using a 5×5×1 **k**-point mesh, rather than simply extracting data from the experiment. This also compensates discrepancies between computational and experimental values, and the obtained value of −6.45 eV/atom was used in the comparison for consistence, even if slightly overbinding compared to the experimental value of −5.81 eV/atom for α-rhombohedral boron bulk.^71^ Furthermore, the catalyst stability can be assessed by calculating the formation energy, *E*_f_, taking bulk Ti, graphite, O_2_, and H_2_ as constituent reactants in their standard state, Ti and graphite being computed likewise as done for B bulk.

### 2.4 Thermodynamic Approach to the Limiting Potential

To provide a comprehensive picture of the NRR mechanism, we rely on the well-known thermodynamic approach as proposed by No̷rskov *et al*.,^72^ widely used in previous studies related to electrocatalysis by MXenes.^21,73−75^ Within this approach, the focus is on the Gibbs free energy differences between different reaction intermediate states, which helps to determine the required limiting potential, *U*_L_, of the reaction. Strictly speaking, every CPET step involves a transition state which can be approximated, as shown by Exner,^76^ and applied to the hydrogen evolution reaction (HER) on the V_2_C MXene.^77^ However, extensive evidence shows that relevant information can be extracted by relying on the thermodynamic picture only.^63,78^

Within the thermodynamic approach, one relies on the free energy profiles which, in turn, requires to first make an estimation of the total adsorption energies of the intermediate species, *E*_ads_^*i*^, obtained as;
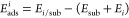
8Here, *E*_sub_ and *E*_*i*/sub_ are the total energy of the corresponding MXene model, featuring a variety of mixed surface terminations, as shown in Figure 1, without and with the adsorbed *i*-species, respectively, and *E*_*i*_ is the total energy of the *i* adsorbed species in vacuum. With this definition, the more negative the *E*_ads_^*i*^, the stronger the adsorption is.

To estimate the reaction of Gibbs free energy change, Δ*G*, at each electrochemical step, we rely on the aforementioned computational hydrogen electrode (CHE) model,^72^ assuming that under standard equilibrium conditions of *pH* = 0, *U* = 0 V, temperature, *T*, of 298.15 K, and a partial pressure of H_2_, *p*_H_2__, of 1 bar, the chemical potential of a pair of H^+^ and e^–^ can be correlated to that of H_2_ at 0 V *vs* the reversible hydrogen electrode (RHE), as;
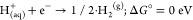
9

Keeping in mind that the chemical potentials or Gibbs free energies of the initial states, H_(aq)_^+^ + e^–^, and the final state, 1/2·H_2_^(g)^, are equal, it turns out that the Gibbs free energy of the proton–electron pair is just half the free energy of the hydrogen molecule. Thus, one can calculate Δ*G* for any elementary reaction step as;
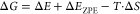
10where Δ*E* is the total energy change of the electrochemical step; Δ*E*_ZPE_ is the change in zero point energy change between initial and final states of this step, estimated from the calculated harmonic frequencies; and Δ*S* is the corresponding entropy change which for adsorbed species involves the vibrational partition function only —explicit formulas for ZPE and Δ*S* can be found in the literature.^21,75,79^ The entropy of gas phase N_2_, H_2_, and NH_3_ species has been sourced from the National Institute of Standards and Technology (NIST) webbook.^80^ Note that the Δ*G* values obtained from eq 10 correspond *pH* = 0 and *U* = 0 V, while Δ*G* values at finite *pH* and *U* can be easily derived as well, as detailed in the literature.^78,81^

From the Gibbs free energy profiles of a given reaction, it becomes possible to evaluate the reaction limiting potential, denoted as *U*_L_, defined as the minimum potential required for a particular electrochemical reaction to proceed successfully under specified reaction conditions.^72^ In the context of NRR, *U*_L_ signifies the electrochemical potential at which each elementary electrochemical hydrogenation step in the reaction becomes exergonic, indicating the minimum energy input necessary for the reaction to advance successfully. The descriptor Δ*G*_max_ is defined as the largest free energy difference between initial and final state for each concerted proton–electron transfer (CPET) step. Computed at *U* = 0 V, is employed here to extract *U*_L_ for NRR, as;
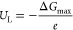
11where generally, the lower the *U*_L_ —the closer to zero overpotential— the higher the reaction activity.

### 2.5 Adsorption Rates

As aforementioned, under reaction condition, N_2_^(g)^ adsorption is a necessary occurrence, while desorption of NH_3_^(g)^ is likely to be fostered by dissolution as NH_4_OH_(l)_. To include the N_2_^(g)^ adsorption necessary step, we rely on kinetic phase diagrams (KPD), as introduced in previous studies.^82,83^ These require a thorough evaluation comparing the molecular adsorption/desorption rates, *r*_ads_ and *r*_des_, respectively, under different temperatures and partial pressures. This allows one to determine the critical turning points at which the adsorption and desorption rates reach equilibrium. The adsorption rates are estimated from nonactivated collision theory, while desorption rates are gained using transition state theory (TST), assuming latest transitions states. Details on the employed formulas are well detailed in the literature.^84^

## 3 Results and Discussion

### 3.1 Models and Stability of B-Based MXenes

First, a systematic sampling search was carried out to investigate the four highly symmetric adsorption sites, including top (T) and bridge (B), hollow carbon (H_C_), and hollow metal (H_M_) of pristine Ti_3_C_2_, as shown in Figure 1a, where H_M_ was found to be always the most favorable adsorption site, consistent with previous studies.^61,85^ In the case of Ti_3_C_2_O, the three possible high-symmetry adsorption sites were also sampled (*cf*. Figure 1b), being H_C_ site the most favorable adsorption site, closely followed by B_O_ site, being most stable on **a**B–O_2_, **a**B–B_O_-O_3_, and **a**B–B_O_-O_4_ (*cf*. Figure 1). When having B_2_ dimers, see Figure 1k,l, a semibridge situation is found, with a molecular display resembling that of ethene.

As far as the stability of each model is concerned, the calculated *E*_ads_, *E*_diff_, and *E*_f_ values are listed in Table S1 of the SI. Note that all the models display a negative formation energy per atom, ranging from −0.37 eV/atom for **s**B–OH to −0.54 eV/atom for **a**B–O, implying that all the studied models are stable with respect to their elemental components. Aside, the B adsorption energies can be also substantial, ranging from −2.53 eV for **s**B–O to −7.66 eV for **a**B–O. As expected, the more negative the *E*_f_, the more negative the *E*_ads_ is. Interestingly, the structural stability is apparently related to changes in the boron arrangement and termination. For the B-substituted (**s**B) models, the stability gradually increases with decreasing number of −O groups and concomitant increasing number of −OH groups, while for B-adsorbed (**a**B) cases, the stability gradually decreases. In addition, the adsorption site and atomic coordination have a certain impact on the stability, since B on H_C_ sites is usually more stable than on B_O_ sites. Increasing the amount of boron (B) as in the explored dimers leads to less stable systems. More importantly, few cases display *E*_ads_ larger than the B bulk cohesive energy, this is, negative *E*_diff_ values. In particular, **a**B–O, while **a**2B–O *E*_diff_ value is close to zero, followed by **a**B–H_C_-O_4_ and **a**B–H_C_-O_3_ with relatively lower *E*_diff_ values. It is clear that B adsorption is favored by a full or large coverage of O adatoms.

The Bader charge of the different compound parts is also listed in Table S1 of the SI. Generally, the Ti_3_C_2_ MXene donates electrons to the T_*x*_ groups, being either −O or −OH, given their larger electronegativity, and in accordance with previous reports,^75^ and charge density difference (CDD) plots in Figure S1 of the SI. As far as B is concerned, it becomes slightly reduced on **s**B models yet slightly oxidized in **a**B ones, a trend that becomes more significant as the ratio of −O groups increase and accentuated on H_C_ conversely to B_O_ sites. In the case of **s**B models, their substitution to −O or −OH implies a certain electron density maintenance to better embed in the T_*x*_ layer. In the case of **a**B situations, the B atoms are slightly positively charged. In fact, for the most stable aforementioned situations, **a**B–O and **a**B–H_C_-O_4_, even **a**B–H_C_-O_3_, the B atom gets its maximum charge of +0.24 *e*, while on the B_2_ dimer of **a**2B–O, the charge per B atom is +0.16 *e*.

Aside, CDD plots shown in Figure S1 of the SI visually confirm the aforementioned results, in that Ti_3_C_2_ donates electron density to −O and −OH terminating groups and B and **s**B situations, while for **a**B and even **a**2B situations, the B atoms become positively charged. Before discussing NRR, the conductivity of these materials should be considered, since a semiconductor-like band gap would handicap electron transfer.^86^ To address this issue, the density of states (DOS) and projected density of states (PDOS) were gained and are shown in Figure S2 of the SI. Briefly, these reveal that all systems exhibit a metallic behavior with the participation of Ti *d* orbitals; C, B, T_*x*_ = O, OH p orbital; and H s orbitals close to the Fermi level. Notice that **s**B and **a**B exhibit significant differences, the former with limited interaction with atomic orbitals from other elements, while the latter involves a strong, covalent mixing with the p orbitals of −O groups, as shown in the −6 to −10 eV region.

### 3.2 N_2_ Adsorption

It has been pointed out^87^ that surface doping with B can enhance the activation of CO_2_ adsorption, since C atoms of adsorbed CO_2_ gain more electrons on B-doped MXene. This trend should also be applicable to N_2_, given the structural and property similarities between the CO_2_ and N_2_ molecules. Furthermore, experimental results have also shown^88^ that the NH_3_ generation rate of B-doped Ti_3_C_2_ is significantly higher than that in the nondoped cases. Thus, here, B-doped MXene is expected to facilitate NRR and surpass in activity of those nondoped scenarios.

**Figure 2:**
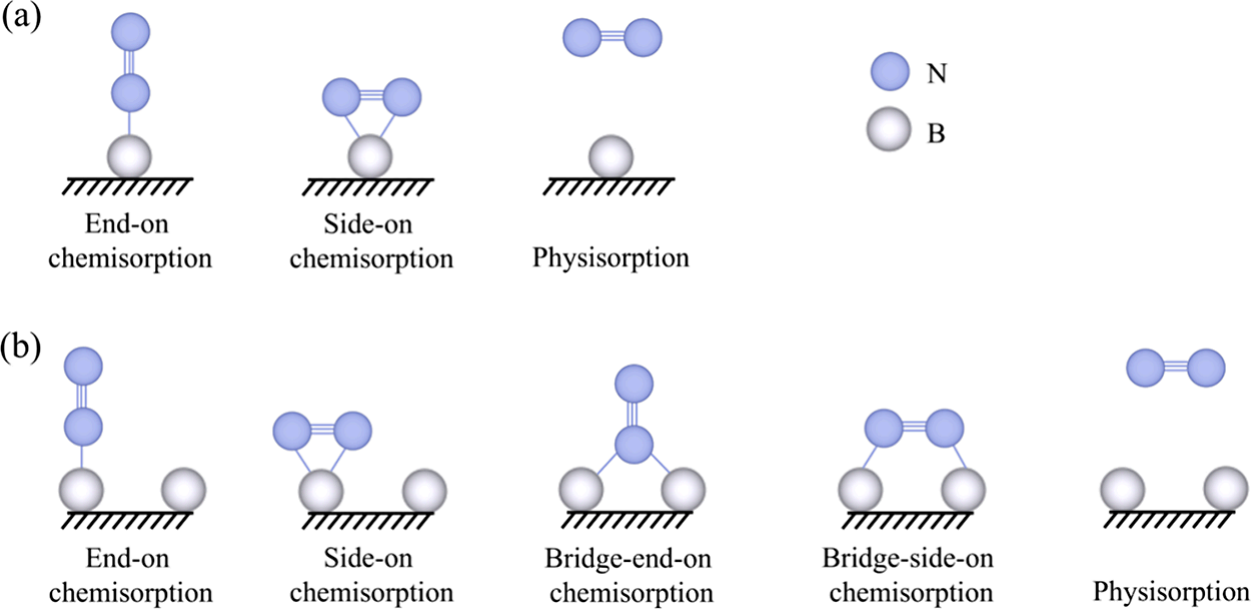
Various adsorption configurations for N_2_ adsorption, including chemisorption and physisorption for (a) single boron and (b) B_2_ dimer models, as shown in Figure 1.

The N_2_ adsorption is a *sine qua non* requirement for the NRR, and one can envisage physisorbed and chemisorbed situations. In the first case, the interaction between doping B and N_2_ is almost negligible, with essentially no charge transfer, whereas for chemisorption, *end-on* and *side-on* structures can be found,^89^ as well as *bridge* configurations^67^ (see Figure 2). The optimized geometries for each conformation are shown in Figure S3 of the SI. Additionally, we also considered adsorption at a single B active center, either **a**B or **s**B, as well as on B_2_ dimers. The N_2_ adsorption energies, *E*_ads_, N_2_ Bader charges, *Q*_N_2__, and closest bond lengths between surface B and N_2_, *d*(BN), and of N_2_ molecule, *d*(NN), are reported in Table S2 of the SI.

First of all, the physisorbed (-*p* states) are easily recognized by small *E*_ads_ values ranging from −0.09 (**a**B–O*-p*) to −0.30 eV (**a**2B–OH*-p*), with essentially no charge transfer between the catalyst and the physisorbed N_2_ molecule. The *d*(BN) distances are large, from 3.26 (**a**B–O-*p*) to 3.51 Å (**a**2B–OH*-p*), and the *d*(NN) distance remains always 1.12 Å; the value of the gas phase molecule is calculated in vacuum. When it comes to the chemisorbed identified states, the interaction between N_2_ and an active B center may involve σ donation from N_2_ or B back-donation to the empty 2π* molecular orbital of N_2_, as suggested in the literature.^90,91^ Compared to **a**B, **s**B exhibits significantly stronger N_2_ adsorption ability, with adsorption energy ranging from −1.43 (**s**B–O-*s*) to −2.79 eV (**s**B–OH-*e*), quite in the line of the reported inherent N_2_ affinity of pristine MXenes.^20^ However, **a**B situations display less strong chemisorption, with adsorption energy values ranging from −0.37 (**a**B–B_O_-O_4_*-s*) to −1.19 eV (**a**B–O_2_*-s*).

The difference toward N_2_ interaction between **a**B and **s**B may be attributed to the negative Bader charge of **s**B, as shown in Table S1 of the SI, easing the back-donation of electron density to the N_2_ 2π* orbital, also in line with the negative Bader charges on N_2_ for **s**B, *Q*_N_2__, from −0.08 (**s**B–O-*e*) to −0.20 *e* (**s**B–OH-*s*), see Table S2 of the SI, and mirrored by a positive charge change on B atoms, Δ*Q*_B_, see Table S2 of the SI. This charge change is also present on **a**B, but to a lesser extent, with *Q*_N_2__ values ranging from −0.07 (**a**B–B_O_-O_4_*-e*) to −0.18 *e* (**a**2B–OH*-bs*). For the chemisorbed states, one finds, as expected, relatively small *d*(BN) bond lengths, from 1.35 (**s**B–OH-*e*) to 1.62 Å (**a**2B–O*-be*), and slightly elongated N_2_ bonds, from 1.16 (**s**B–O-*e*) to 1.40 Å (**s**B–OH-*s*). Clearly, the exploration yielded a handful of chemisorbed minima prone to N_2_ activation and posterior reduction.

Furthermore, the analysis also suggests that −OH environment and the *side-on* adsorption mode strengthen the N_2_ interaction on **s**B models, while for **a**B, when B is adsorbed on the H_C_ site, its three *sp*^3^ hybridized orbitals and three electrons are used for −O coordination, leaving one empty *sp*^3^ orbital as possible acceptor, and not prone to back-donation, which explains why these sites lead to physisorption. For **a**2B dimers, *bridge*-*side-on* maximizes the interaction with N_2_, where each B has one free electron to bond each of the N atoms in the N_2_ molecule, ultimately weakening the molecular bond. Thus, the donation and back-donation mechanism, the B electron charge, and the coordination mode freeing sp^3^ electrons are found to be key in N_2_ adsorption and activation.

To further confirm the existence of a direct bond between B doping atoms and the N_2_ molecule, insights are withdrawn from the minima PDOS, as shown in Figures S4–S6 of the SI. The plots for the physisorbed state show no significant overlap between B and N_2_*sp*^3^ orbitals, where **a**B–H_C_-O_4_*-p* is a clear example. The opposite occurs for the chemisorbed states with clear orbital overlap (see *e.g.*, the cases of N_2_ on **s**B–O-*s* or **a**B–B_O_-O_4_-*s*) with localized discrete states with contributions from both B and N atoms, in line with covalent-like bonds. The charge density differences (CDD), shown in Figure S7 of the SI, are in line with the mentioned donation–acceptation picture.

**Figure 3:**
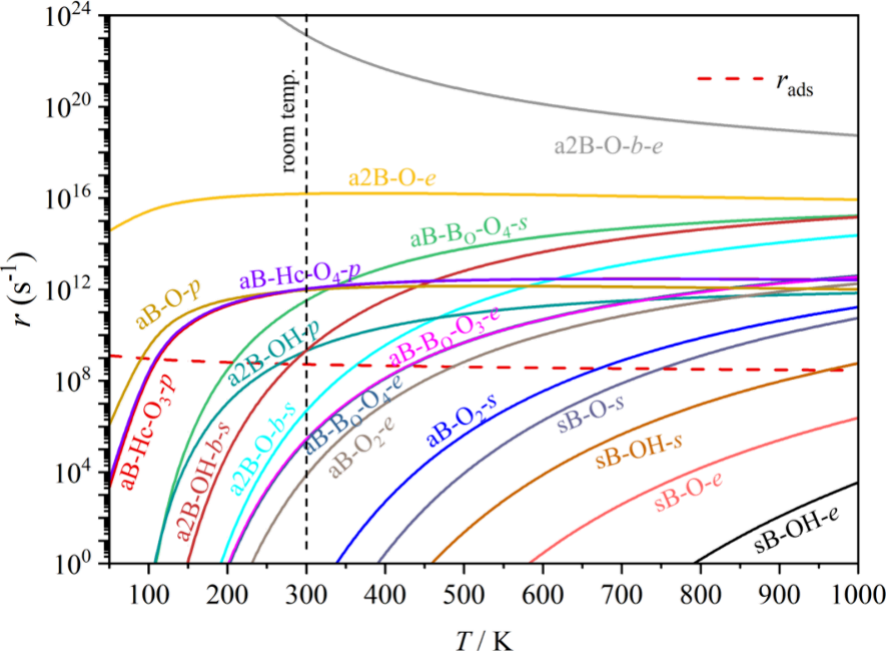
Calculated adsorption, *r*_ads_, and desorption, *r*_des_, N_2_ rates on studied models are shown in Figure S5 of the SI, as a function of temperature, *T*, and the gas partial pressure, *p*, here shown for 1 bar. Red dashed line represents *r*_ads_ for physisorption.

Last but not least, to go beyond the static adsorption picture and to inspect whether N_2_^(g)^ would get adsorbed on the catalyst, we compared the adsorption and desorption rates, *r*_ads_ and *r*_des_, respectively, for all models and adsorption modes, much following the employed procedure used in the past to acquire the KPD.^92,93^ As seen in Figure 3, under reaction working condition of *T* = 300 K and 1 bar of N_2_^(g)^ partial pressure, a number of models and sites feature an adsorption rate superior to the desorption rate, in particular **a**2B–O-*bs*, **a**B–B_O_-O_4_-*e*, **a**B–B_O_-O_3_-*e*, **a**B–O_2_-*e*, **a**B–O_2_-*s*, **s**B–O-*s*, **s**B–OH-*s*, **s**B–O-*e*, and **s**B–OH-*e*; thus, in all cases, chemically bound, activated N_2_, with an adsorption energy stronger than −0.67 eV, in concordance with values listed in Table S2 of the SI, and so, underscoring the viability of **s**B modes than **a**B ones. Note that once NH_3_ is formed, it will spontaneously desorb as NH_4_^+^ frees the active sites. Also since NRR typically takes place in aqueous solution where the produced NH_3_ reacts with H_2_O_(l)_ to form NH_4_OH_(l)_, there is no need to consider the adsorptive and desorptive landscape for NH_3_^(g)^.

By correlating the N_2_ adsorptive capacities and doping model stabilities, the only model with a priori kinetic stability capable of adsorbing and activating N_2_^(g)^ would be the B dimer on O-terminated MXene, the **a**2B–O, see Table S1 of the SI and plots in Figure 3. Still, the NRR Gibbs free energy profile will be studied for other close models, since such systems could be kinetically metastable, and to capture trends and mechanism variations with respect to the model composition (see below).

### 3.3 NRR Mechanisms

**Figure 4:**
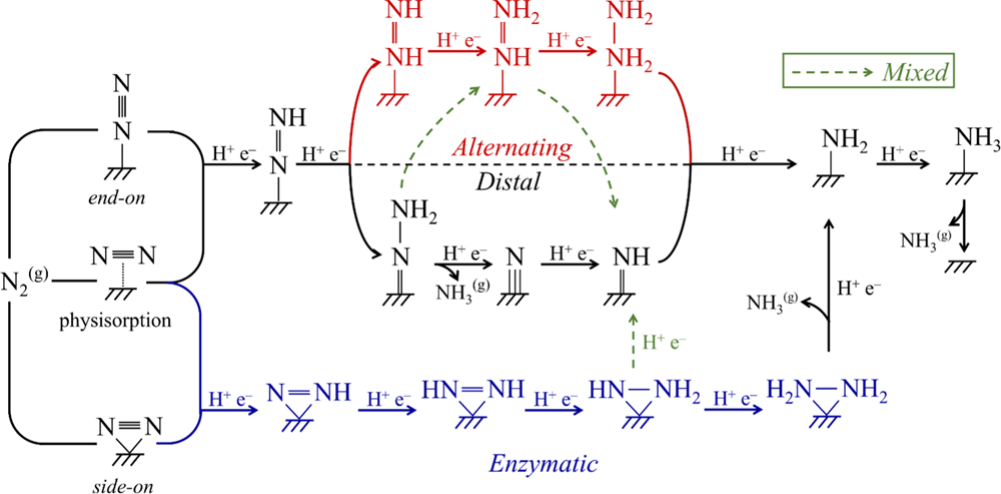
Schematic diagram of possible reaction pathways for NRR on B-based Ti_3_C_2_ MXene. Black, red, and blue solid arrows represent the *distal*, *alternating*, and *enzymatic* pathways, respectively. Green dashed arrows represent the *mixed* pathway.

Before delving into the NRR free energy profiles, it is mandatory to define the possible mechanisms and to keep in mind the possible competition with the HER.^94^ Previous research suggested that NRR predominantly occurs through *distal* and *alternating* mechanisms in *end-on* or *enzymatic* mechanism in *side-on* adsorption modes,^95^ as illustrated in Figure 4. However, in the present study, apart from the three mechanisms, physisorption is also considered as a possible step. The *distal* mechanism has its name since it assumes that the CPET first attacks the N atom farthest from the catalyst surface and continues in subsequent CPET steps until a first NH_3_^(g)^ is synthesized and released, leaving a N* moiety on the catalyst surface, which gets later fully reduced until the second NH_3_^(g)^ is gained. At variance, in the *alternating* mechanism, the CPET alternate in between the two N atoms of the *end-on* situation. Finally, the *enzymatic* mechanism mimics biological mechanisms of N_2_ fixation, where one departs from an activated *side-on* adsorption mode, favoring an alternating pattern of CPET for the two N atoms.

Before focusing on the reaction free energy profiles, it is worth stating here that in the case of **a**2B exhibiting dual active sites, the NH_2_NH_2_* does not appear in the *enzymatic* mechanism since optimizations consistently showed that the N–N bond breaks during the hydrogenation of NHNH_2_*, directly forming two NH_2_* species. Furthermore, *mixed* situations can occur, where NHNH_2_* is formed when reducing NNH_2_* instead of gaining NH_3_^(g)^ and N* adatoms through the *distal* pathway (see Figure 4), or when, in the *enzymatic* path, the NHNH_2_* hydrogenates to NH* and NH_3_^(g)^ instead of forming NH_2_NH_2_*. Thus, these mixed paths connecting distal with alternating or enzymatic paths are also regarded in the following.

### 3.4 NRR Reaction Free Energy Profiles

**Figure 5:**
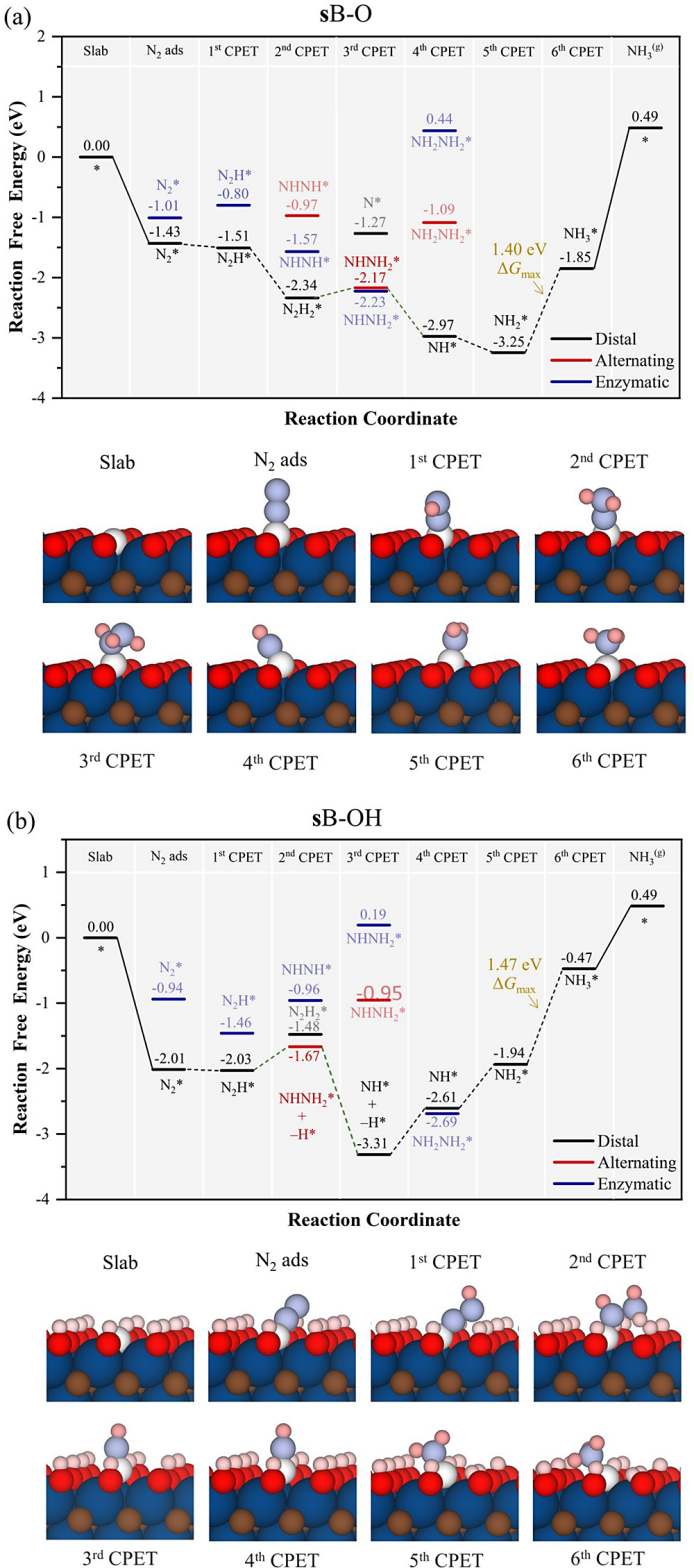
Gibbs free energy, Δ*G*, diagrams of NRR on (a) **s**B–O (top), and (b) **s**B–OH (bottom), under standard working conditions of *T* = 300 K, partial gas pressures of 1 bar, *pH* = 0, and *U* = 0 V. Solid lines represent chemical steps such as N_2_^(g)^ adsorption or as-generated NH_3_^(g)^ desorption, while dashed lines represent CPET steps. −H* notation implies the H transfer from the surface −OH group. Below the reaction paths, side views of the atomic models for the different reaction steps are shown. N and B atoms are shown in light blue and white color, respectively; the H atoms of −OH group and the proton of CPET are represented by light pink and dark pink, respectively, while the rest of the color coding is shown in Figure 1. Dark numbers and symbols indicate the optimal path, while light ones indicate nonoptimal.

Let us finally analyze the three possible mechanisms for the electrochemical reduction of N_2_ into NH_3_, with the ultimate aim of determining the most favorable pathway, while investigating the influence of B doping site, **a**B *vs***s**B, having B single atoms or dimers, *i.e.*, **a**B *vs***a**2B, and the effect of nearby functional groups. To this end, we start with substitutional B situations (**s**B), with surface −O or −OH groups, as shown in Figure 5. In these models and as mentioned above, a prominent feature is the exceptionally strong N_2_ adsorption capability. On **s**B–O, except for the NHNH_2_* and final NH_3_* generation steps, the other CPET are exergonic. Indeed, Δ*G*_max_ corresponds to the final CPET with a value of 1.40 eV as the potential-determining step (PDS), and the most favorable pathway is *mixed,* mainly following *distal* mechanism, except for the NHNH_2_* formation which correspond to *alternating* one. On the **s**B–OH model, the NH_2_* → NH_3_* is also the PDS with Δ*G*_max_ of 1.47 eV and also follows the *distal* mechanism, except for the generation and hydrogenation of NHNH_2_* species, which follows the *mixed* route to generate NH* and NH_3_* directly. Here, at variance with **s**B–O, all steps are endergonic or in equilibrium except for the NHNH_2_* hydrogenation step. Note that, as observed in, e.g., CO_2_RR on Ti_3_C_2_T_*x*_ models,^75^ the surface, −OH groups can transfer H atoms and that is found in the *alternating* path when reducing N_2_H*, where NHNH_2_* is formed; at the same time, one vicinal −OH group transfers its H to form this moiety (see Figure 5), being a much more stable intermediate. Still, accounting for energetic preferences on path bifurcations, the *distal*-*alternating mixed* mechanism is, in principle, preferred.

**Figure 6:**
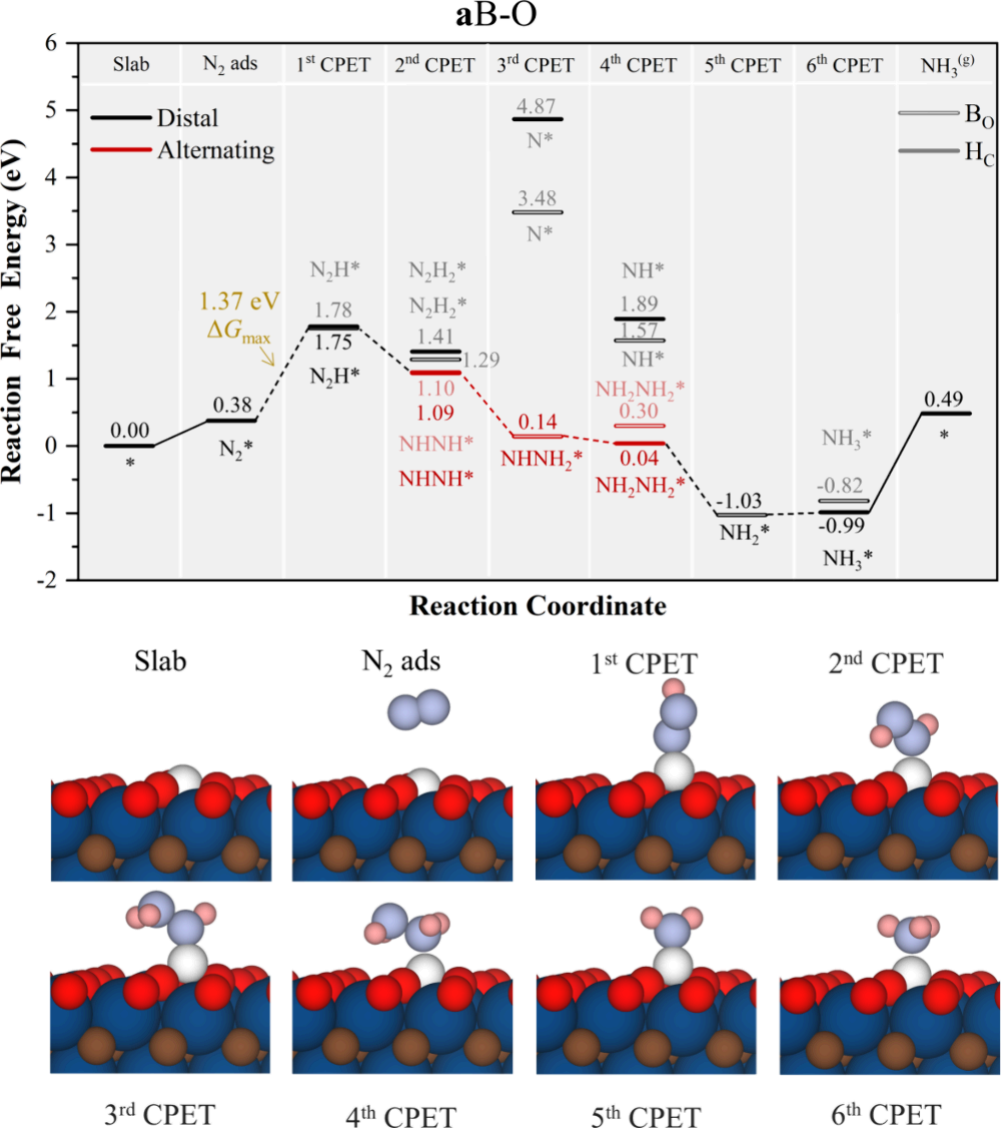
Gibbs free energy, Δ*G*, diagrams of NRR on **a**B–O under standard working conditions of *T* = 300 K, partial gas pressures of 1 bar, *pH* = 0, and *U* = 0 V. Solid lines represent chemical steps such as N_2_^(g)^ adsorption or as-generated NH_3_^(g)^ desorption, while dashed lines represent CPET steps. Below the reaction paths, side views of the atomic models for the different reaction steps are shown color-coded as in Figure 5.

In the situations with adsorbed B, **a**B, one may have B on bridge sites, B_O_, or hollow sites, H_C_, and the reaction may imply changes in the adsorption mode. In the case of **a**B–O (see Figure 6), the B atom is on a H_C_ site (see Figure 1). There the reaction sequence after N_2_ physisorption leads to N_2_H*, the PDS with a Δ*G* of 1.37 eV, where situations having B at H_C_ or B_O_ are nearly equivalent. From there on, the reaction primarily follows the NHNH* *via* the *alternating* mechanism, alternating also B being between H_C_ and B_O_. This is because when the H addition implies a N–N bond breaking, the freed lone pair is used to make a new covalent bond with B, at the expense as well of breaking a B–O bond, and adopting a B_O_ conformation. By adding a new H bond, the generated extra B–N bond gets broken to use the N lone pair in the new N–H bond, and then, B goes back to a H_C_ conformation, maximizing bonds with surface O atoms.

**Figure 7:**
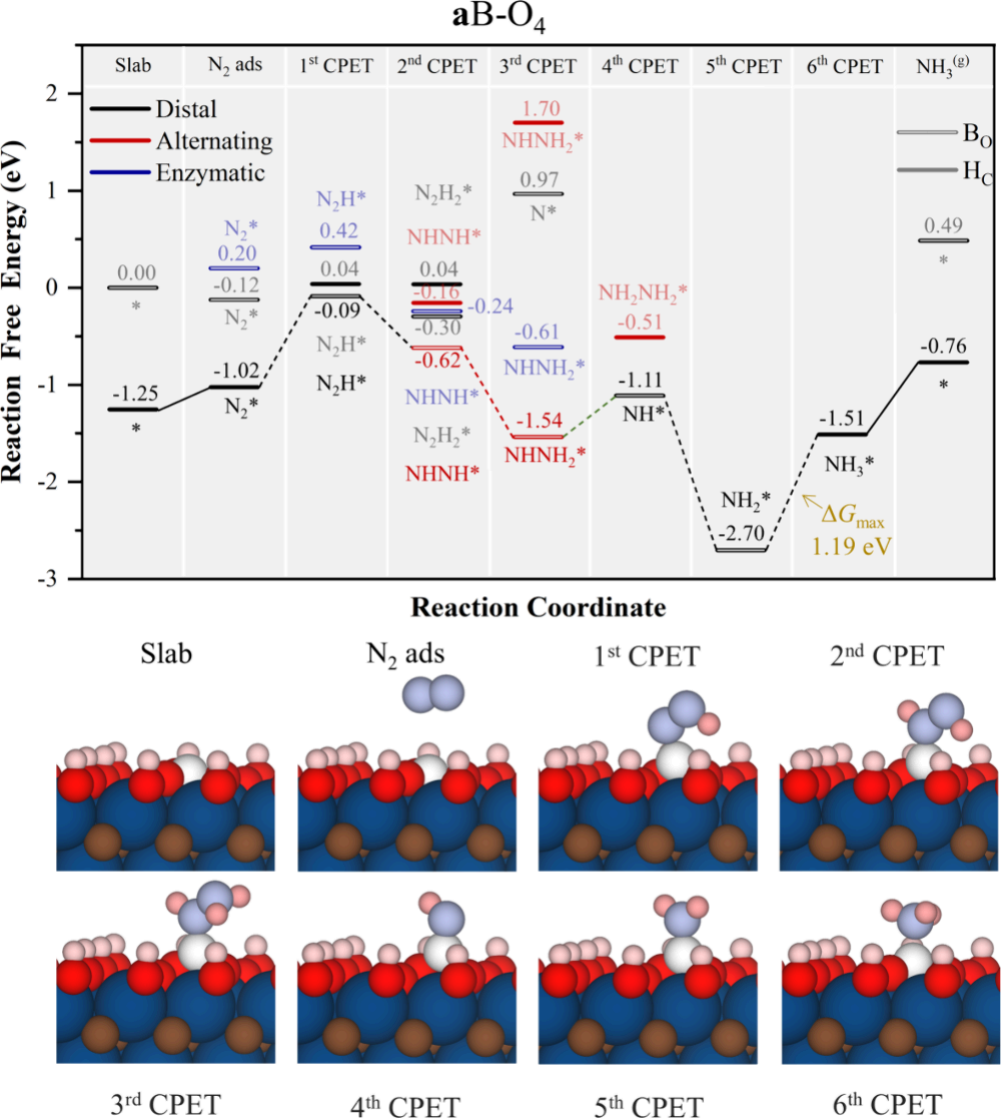
Gibbs free energy, Δ*G*, diagrams of NRR on **a**B–O_4_ under standard working conditions of *T* = 300 K, partial gas pressures of 1 bar, *pH* = 0, and *U* = 0 V. Solid lines represent chemical steps such as N_2_^(g)^ adsorption or as-generated NH_3_^(g)^ desorption, while dashed lines represent CPET steps. Below the reaction paths, side views of the atomic models for the different reaction steps are shown color-coded as in Figure 5.

The next model with highest concentration of −O groups is the **a**B–O_4_, with ^4^/_9_ of the surface being −O groups and the rest −OH groups. The NRR free energy reaction profile is shown in Figure 7, which departs from B in H_C_ as this is the more stable situation (see Table S1 of the SI). Here, a first step that is costly is the first CPET to form N_2_H* on a B_O_ mode, with a Δ*G* of 0.93 eV. However, after keeping B in B_O_ and following the *mixed* pathway, the PDS is on the last NH_3_* formation, similar to the **s**B models, with a Δ*G* of 1.19 eV.

**Figure 8:**
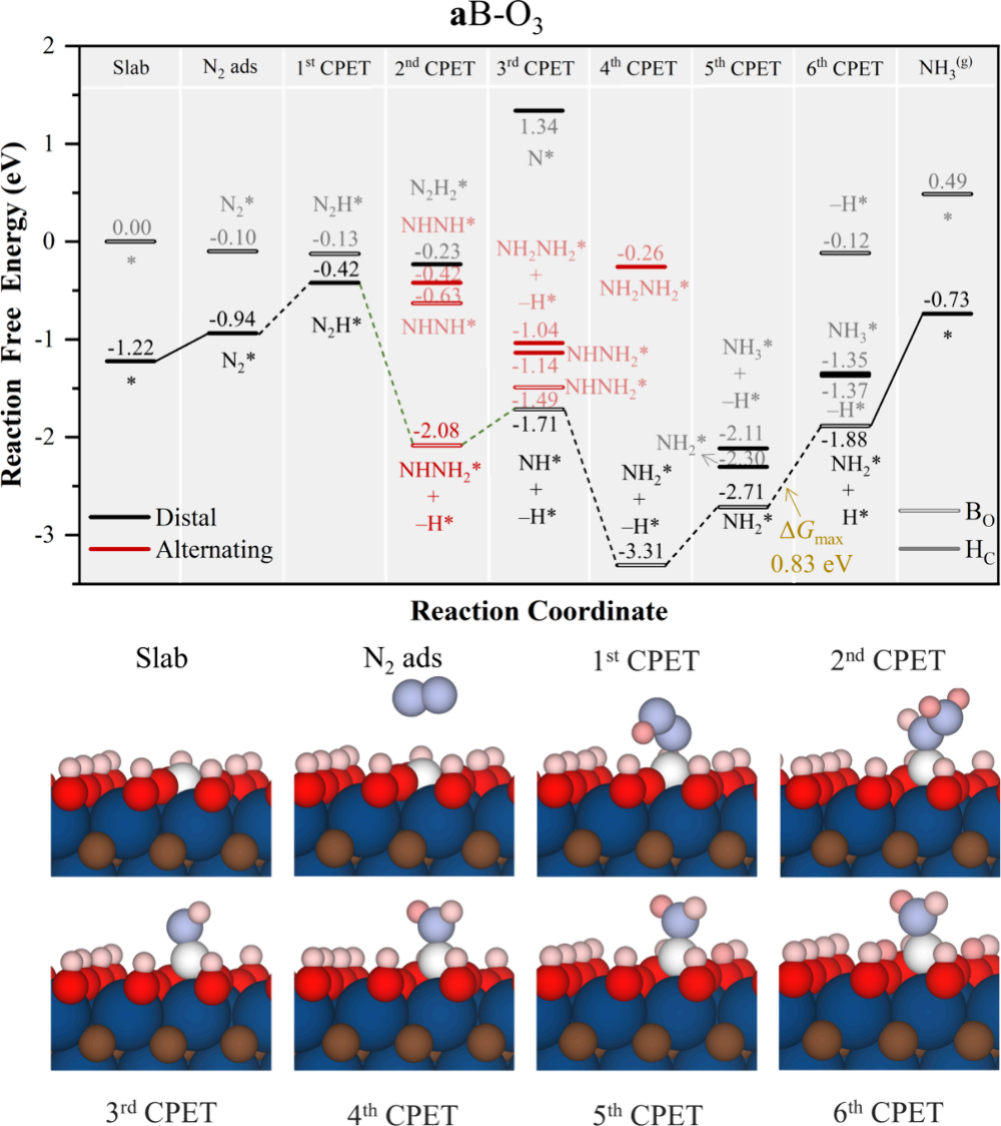
Gibbs free energy, Δ*G*, diagrams of NRR on **a**B–O_3_ under standard working conditions of *T* = 300 K, partial gas pressures of 1 bar, *pH* = 0, and *U* = 0 V. Solid lines represent chemical steps such as N_2_^(g)^ adsorption or as-generated NH_3_^(g)^ desorption, while dashed lines represent CPET steps. Below the reaction paths, side views of the atomic models for the different reaction steps are shown color-coded as in Figure 5 and notation as in Figure 5.

The situation changes, though, when increasing the number of −OH groups nearby that can participate in the reaction. This is visible in the **a**B–O_3_ model in Figure 8. The initial free energy profile is similar to that of **a**B–O_4_, departing from the H_C_ site for B, up to the formation of N_2_H* with a Δ*G* of 0.52 eV. From this point on, the further hydrogenation through *distal-alternating mixed* path implies the simultaneous transfer of one H from a vicinal −OH group to form NHNH_2_*, being this step quite exergonic by −1.66 eV. From this point on, the next CPET implies the formation of the first NH_3_* leaving behind NH*, endergonic by 0.37 eV, and the subsequent CPET on NH* to form NH_2_*, again quite exergonic by −1.6 eV. At this point, the as-generated −O can be hydrogenated to recover the former −OH group with a Δ*G* of 0.60 eV, and the formation of −OH group from the preexisting −O group becomes the costliest CPET and thus constitutes the PDS with a Δ*G* of 0.83 eV. From this point, the reaction ends up with the second NH_3_ molecule formation.

**Figure 9:**
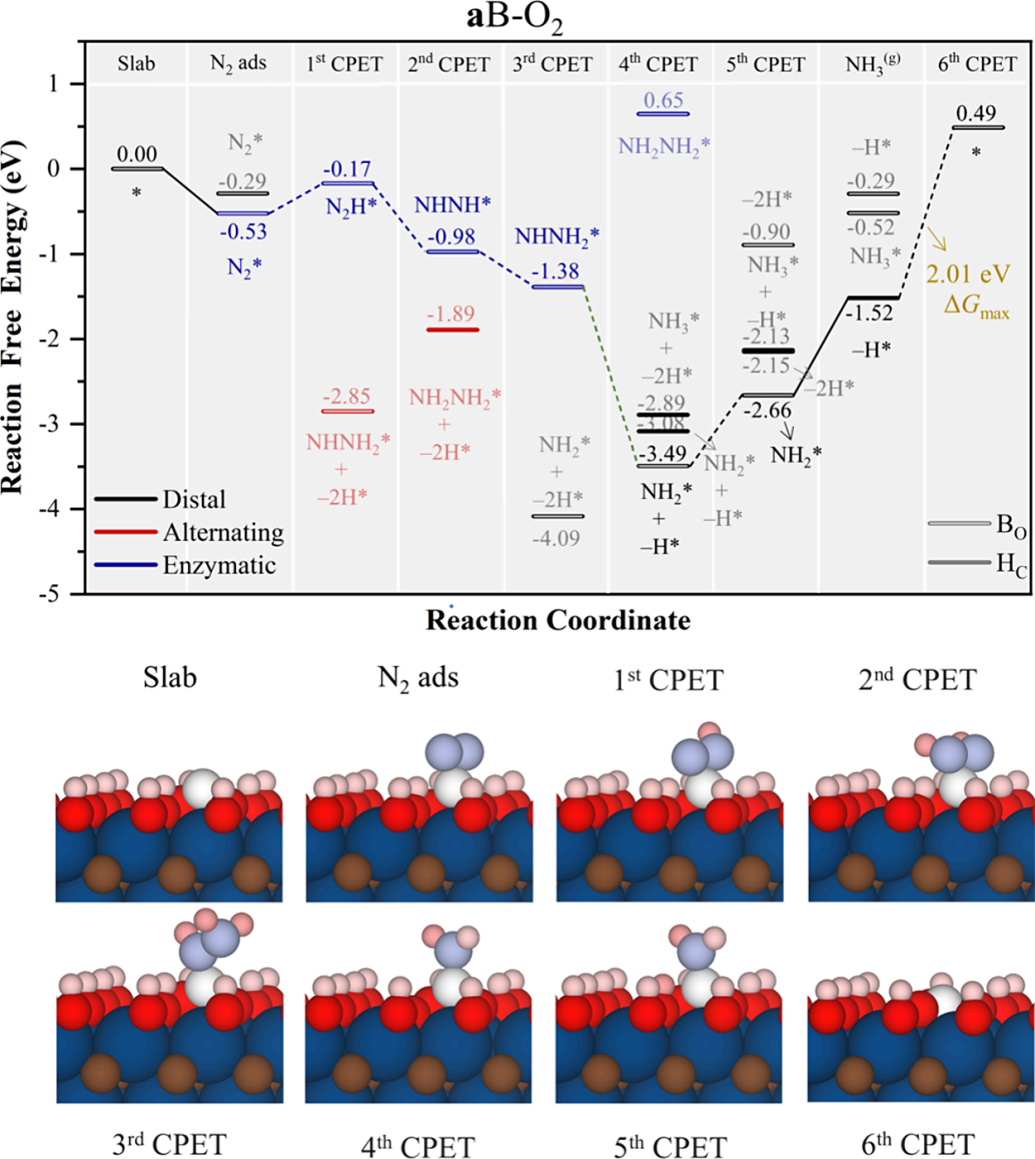
Gibbs free energy, Δ*G*, diagrams of NRR on **a**B–O_2_ under standard working conditions of *T* = 300 K, partial gas pressures of 1 bar, *pH* = 0, and *U* = 0 V. Solid lines represent chemical steps such as N_2_^(g)^ adsorption or as-generated NH_3_^(g)^ desorption, while dashed lines represent CPET steps. Below the reaction paths, side views of the atomic models for the different reaction steps are shown color-coded as in Figure 5 and notation as in Figure 5.

Finally, on the **a**B–O_2_ model, the reaction proceeds mostly with B on B_O_ site, as expected (see Figure 9). N_2_* initially follow the *enzymatic* pathway, and further hydrogenation from NHNH_2_* after the first three CPET has a very stable jump toward NH_2_* placing into the *distal* path, involving one H atom transfer from a vicinal surface −OH groups, with a Δ*G* of −2.11 eV. From this point on, the as-generated −O group is compensated, and next, the second NH_3_ formation is achieved with a Δ*G* of 1.14 eV. After NH_3_ desorption, the final CPET back-recovers the B_O_ site for B and also regenerates a second surface −OH, becoming the PDS with Δ*G* = 2.01 eV. Thus, in general, for large −OH coverage, as in **a**B–O_2_ and **a**B–O_3_, the participation of surface −OH groups is to be highlighted, modulating the reaction profile, since both the PDS involve the −OH participation. However, the results of **a**B–O_2_ indicate that an excessive amount of surface −OH is not necessary, and a moderate amount of −OH can therefore improve the reaction as in **a**B–O_3_. Aside, even if **s**B models exhibit excellent N_2_ adsorption, their performance is generally poorer compared to **a**B models, which seems to benefit from a high surface presence of −OH groups, as seen in **a**B–O_3_ with Δ*G*_max_ of 0.83 eV, the smallest so far.

**Figure 10:**
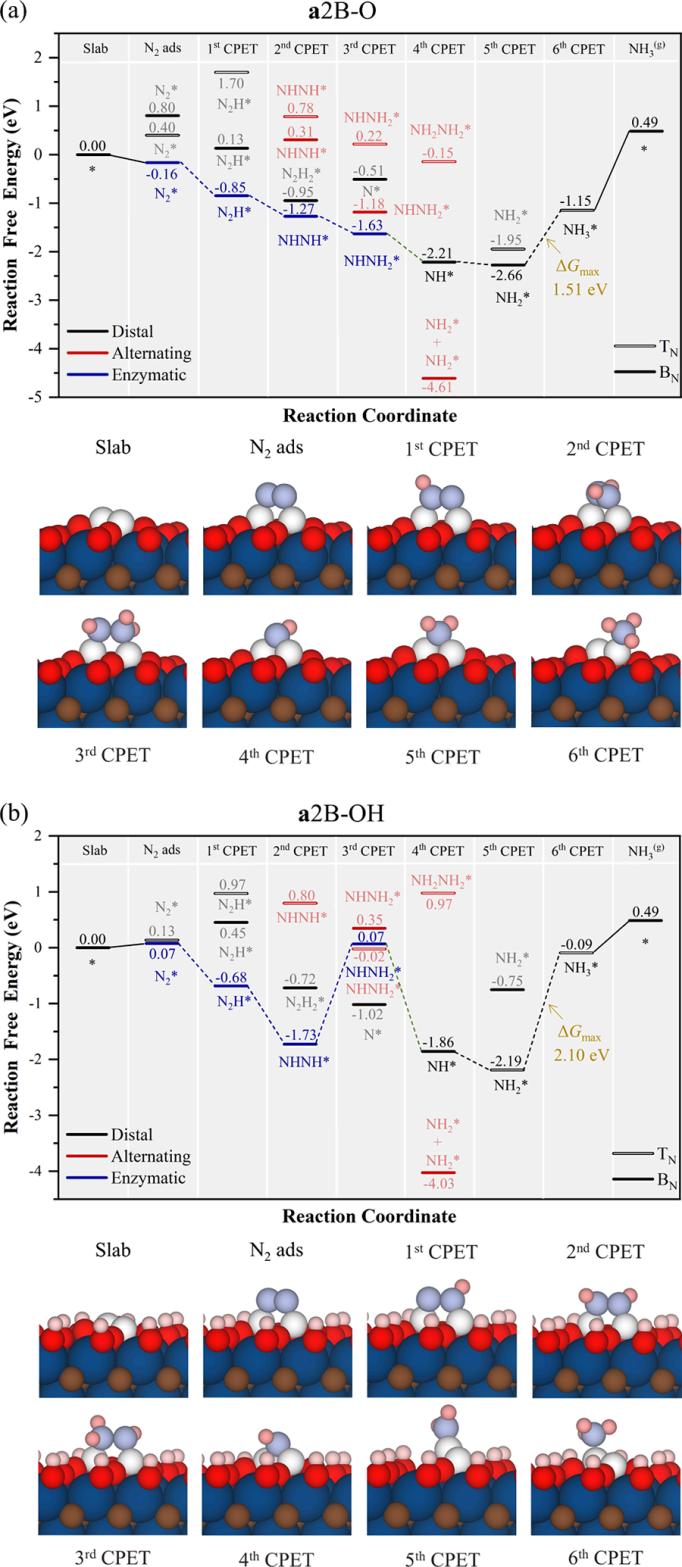
Gibbs free energy, Δ*G*, diagrams of NRR on (a) **a**2B–O (top), and (b) **a**2B–OH (bottom), under standard working conditions of *T* = 300 K, partial gas pressures of 1 bar, *pH* = 0, and *U* = 0 V. Solid lines represent chemical steps such as N_2_^(g)^ adsorption or as-generated NH_3_^(g)^ desorption, while dashed lines represent CPET steps. T_N_ represents adsorption on top of a nitrogen atom, and T_B_ represents adsorption on a bridge between two nitrogen atoms. Below the reaction paths, side views of the atomic models for the different reaction steps are shown color-coded as in Figure 5.

At this point, one may wonder what the effect of having B dimers would be. Compared to **a**B, on double adsorbed B sites (*cf*. **a**2B in Figure 10), two additional N_2_^(g)^ adsorption modes are possible, *be* and *bs*, as shown in Figure 2. These are also viable for hydrogenated species in the course of the NRR. However, since in **a**2B–O *bs,* N_2_ adsorption is faster, as seen in Figure 3, we depart from this mode to generate the reaction free energy profile which is presented in Figure 10, where T_N_ denotes adsorption on top of a nitrogen atom and T_B_ denotes adsorption on a bridge between two nitrogen atoms. The B_2_ surface dimer prompts following the *enzymatic* pathway downhill up to NHNH_2_* and then *mixed* shortcut to get on *distal* to form NH* and NH_2_*, which bounds two B atoms, and finalizes the reaction being formation of last NH_3_* the PDS with a Δ*G* of 1.51 eV. Interestingly, in the **a**2B–OH model, the reaction route is more complex. Despite the slightly more favorable N_2_* *bs*, the high stability of reaction intermediates along the *mixed* path from the *enzymatic* path leads the final NH_3_* formation as the PDS with Δ*G* of 2.1 eV. However, it can be argued that a route in which the bridge and top sites on B_2_ makes the reaction route go through *alternating* path could be possible, as shown in Figure S8 with PDS being the last NH_3_ formation, with Δ*G* of 0.99 eV, but the thermodynamic will be larger when all sites were occupied by the most favorable mechanism.

**Figure 11:**
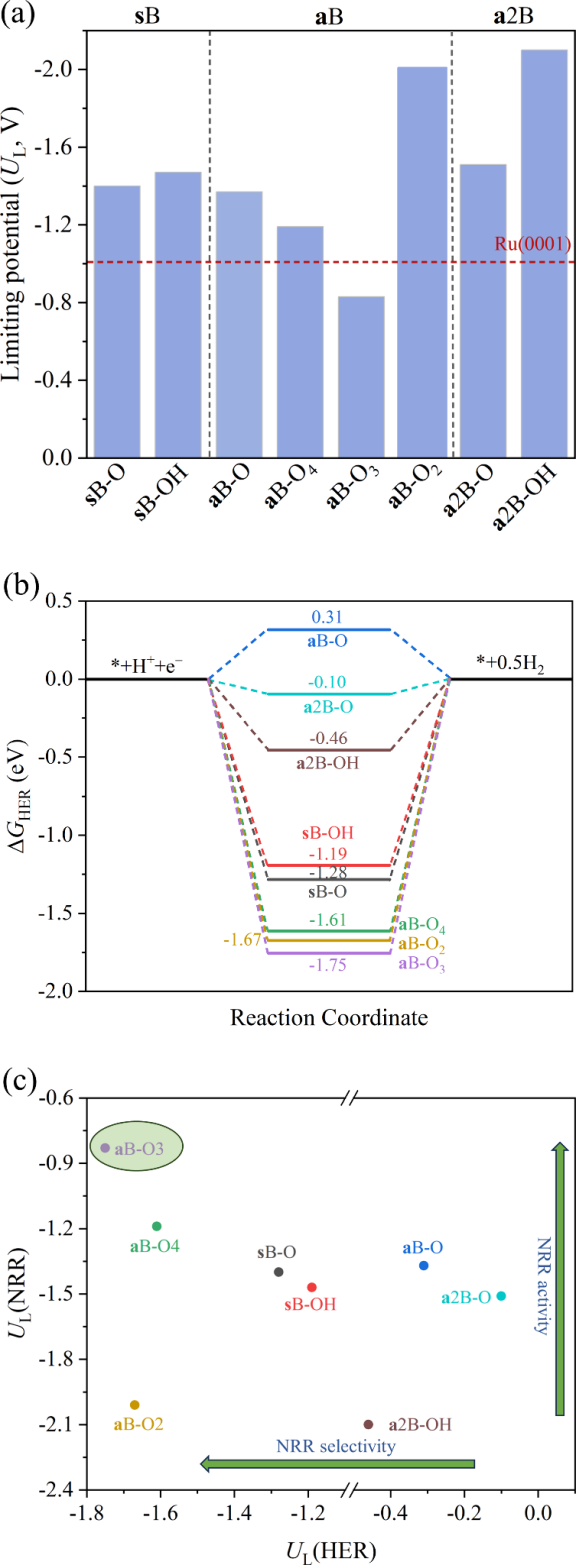
(a) Comparison of *U*_L_(NRR) of the different models with respect to Ru (0001) reference, (b) free energy change for HER on the studied catalysts, and (c) *U*_L_(NRR) *vs U*_L_(HER). Value for Ru(0001) has been taken from ref (96).

In summary, from the studied reaction paths and as summarized in Figure 11a, one can clearly state that (*i*) **a**B generally features slightly smaller *U*_L_ compared to **s**B; (*ii*) having a combination of surface −O and −OH groups also generally reduces the *U*_L_; with (*iii*) the free energy reaction path sometimes affected by the involvement of vicinal surface −OH transferring simultaneously their H atoms; (*iv*) actually having **a**B is best when B being on H_C_ surrounded by −OH; (*v*) *distal*, *alternating*, *enzymatic*, and *mixed* paths are often visited; and (*vi*) finally, having adsorbed B with moderate −OH groups is best for reducing *U*_L_. At this point, still, one has to consider that upon reduction conditions, the NRR will compete with HER, as happens as well with CO_2_RR.^75^ The HER and the selectivity toward NRR and a comparison with the literature is treated in the next section.

### 3.5 Performance and NRR *vs* HER

The present assessment of the overall performance relies on the calculated Δ*G*_max_ value alongside its respective *U*_L_. Figure 11a compares the Boron-doped Ti_3_C_2_T_*x*_ models as NRR electrocatalyst to the Ru (0001) reference of having Δ*G* = −1.08 V,^96^ yet gained using revised PBE functional with no dispersion correction interactions, so one should avoid making clear differences for Δ*G* differences within the DFT standard accuracy of *ca*. 0.2 eV. Note that the static view of the catalytic site here in theoretical model may differ slightly from actual experimental procedure, but the key point is to compare it with the above Ru(0001) studied by No̷rskov and coworkers^96^ based on a thermodynamic model. Electrolyte and solvent effects will for sure stabilize the intermediates through electrostatic and hydrogen bonding, respectively. This implies that including an implicit solvent model would not be adequate. A better description requires adding the solvent explicitly, which can be done, for instance, using a microsolvation model, perhaps including also electrolytes. However, we would argue that in the absence of experimental results carrying out the formidable amount of calculations required is not justified. Furthermore, we aim at a direct comparison to the previous results for NRR on Ru(0001) from No̷rskov and coworkers^96^ using exactly the same models and theoretical approach. We are confident that, in spite of this limitation, the present model can capture the essential trend, and the qualitative conclusions are valid, and we hope that our findings will further inspire experimentalists to NRR under heteroatom doped MXene. In any case, Figure 11a evidences a clear benefit possibly extendable to other MXene compositions. Moreover, the HER is a major competing side reaction with the NRR, which may decrease the faradaic efficiency (FE) in experiments. HER is usually evaluated using a three-state diagram,^21^ which includes the initial CPET to form H*, and a second one which directly generates the H_2_ product. Within this scheme, also treating the B atom as the active site, the closer the absolute value of Δ*G*_max_ of HER is to zero, the closer is the material to the ideal HER catalyst. As shown in Figure 11b, the studied model catalysts perform significantly differently, with **a**2B–O demonstrating excellent HER performance, with |Δ*G*_HER_| of 0.1 eV, while **a**B–O_3_ is worst with a |Δ*G*_HER_| of 1.75 eV (see values in Table S3 of the SI). At this point, one can compare the *U*_L_ of NRR, *U*_L_(NRR) to that of HER, *U*_L_(HER), as illustrated in Figure 11c. Here, the competition between NRR is evident; in some cases, the HER is more easily achieved than NRR on fully −O covered adsorbed **a**B–O and **a**2B–O. However, for adsorbed situations with mixed quantities of surface −O and −OH groups, **a**B–O_3_ and **a**B–O_4_, the NRR is more favored than HER (see Figure 11b). Finally, on substituted **s**B–O and **s**B–OH, and B adsorbed on high −OH covered, **a**B–O_2_, the competition between NRR and HER is more evident. Looking for low *U*_L_ and relatively good performance of NRR vs HER, the **a**B–O_3_ situation would be optimal. Note that strategies exist to inhibit HER, including limiting proton concentration or transfer rate,^97,98^ using nonaqueous proton donors to inhibit hydrogen adsorption on catalysts, and providing protective layers to prevent proton transfer to the surface.^99,100^

Finally, it is worth addressing the experimental synthesis feasibility of the above raised NRR electrocatalyst candidates. The preparation of B-doped Ti_3_C_2_T_*x*_ involves placing the solution into a Teflon-lined autoclave and a hydrothermal treatment at 180 °C for 24 h.^101^ However, previous studies^50,102^ have shown that high-temperature treatment may lead to the removal of surface −OH groups, which poses a challenge in maintaining a high −OH state, if willing to get highly selective **a**B–O_4_, and best performing **a**B–O_3_. Still, previous studies on computational Pourbaix diagrams^81,103^ indicate the stability of surface terminations as a factor of pH and *U*, where pure −O, −OH, and mixed −O/–OH situations are reachable at low *pH* and slightly positive, negative, and close to zero overpotential, respectively, solving stability issues under low *pH* and potential operating conditions. For further verification, under the consideration of only surface terminations −O and −OH, different ratios were explored by constructing a Pourbaix diagram to determine the most stable termination under working conditions of pH and *U*, as shown in Figure S9. The results show that as the potential becomes more negative, the −OH termination becomes more stable. Therefore, the −OH termination is highly stable under NRR working conditions. Furthermore, it is necessary to consider the stability of the B active site. The stability in the electrochemical reaction was assessed considering the following two scenarios: (*i*) only the boron atom and (*ii*) both the boron atom and the −O/–OH termination groups.

For the first scenario, a simple thermodynamic cycle can be used to form boric acid, as shown Scheme S1 of the SI; herein, the equilibrium potential (*U*_diss_) for boron oxidation introduced as a descriptor is listed in Table S1 of the SI, which indicates that B easily dissolves into B(OH)_3_ under reduction experimental condition. However, note that the fully spontaneous reaction with Δ*G* = *nF*Δ*E*° where Δ*E*° = −0.89 V corresponds to the standard electrode potential for B_(s)_ forming B(OH)_3_ indicates that as long as B_(s)_ can form —meaning B can escape the surface— B(OH)_3_ will form. Thus, from purely thermodynamic arguments, these systems are not stable. However, focusing only on the thermodynamic cycle is insufficient since the key issue here is that B active sites need to escape from the surface, and this is a high energy step implying a kinetic constraint for the dissolution reaction so that the systems become metastable.

For the second scenario, the corresponding stability is investigated by Pourbaix diagram, as shown in Figure S10 of the SI, indicating that surface composition has the lowest Δ*G* value under any *pH* and *U* conditions; the details are reported in Section S1 of the SI. These results coincide with the stability analysis described above, *i.e.*, the **a**B–O, **a**B–H_C_-O_4_, and **a**B–H_C_-O_3_ have best stability under specific electrochemistry condition. Moreover, several studies^88,101,104−106^ have confirmed the stable presence of boron in boron-doped Ti_3_C_2_ MXene, identifying Ti–B, B–O, and Ti–B–O bonds. High-resolution transmission electron microscopy (HRTEM), energy-dispersive X-ray spectroscopy (EDS), and X-ray diffraction (XRD) patterns also showed that boron particles were widely distributed on the surface of Ti_3_C_2_ nanosheets and that boron-doped Ti_3_C_2_ remains stable before and after reaction.^88,104^ Additionally, coating MXenes with a protective layer has been recommended to enhance their stability.^101^

## 4 Conclusions

In this study, we investigated the potential performance of B-based Ti_3_C_2_ MXene for the NRR, with a specific focus on different boron configurations, including substituted/adsorbed boron (**s**B/**a**B), the distinct B coordination (H_C_/B_O_), and the number of boron atoms, as well as the impact of different surface termination ratios on catalytic activity, including two **s**B and eight **a**B models. The N_2_ adsorption energy and activation rate was considered to determine whether the catalytic reaction could proceed. Moreover, we also analyzed the involvement of hydrogen atoms from the −OH terminating groups, especially for mixed −O/–OH terminations in **a**B–O_2_ and **a**B–O_3_ models.

The present DFT calculations revealed that, despite **s**B models have the strongest N_2_ adsorption capacity, followed by low-coordinated **a**B, the NRR performance of **s**B is significantly lower than that corresponding to **a**B situations. From the present models, it appears that a moderate number of −OH groups at the catalyst surface, neither excessive nor too low, is better for NRR performance, especially for **a**B–O_3_, which exhibited significantly higher NRR performance than cases fully −O terminated, such as **s**B–O, **a**B–O, and **a**2B–O, and the cases with high −OH-terminated presence **a**B–O_2_ and **a**B–O_4_ with low −OH coverage. In addition, **a**B–O_3_ is predicted to outperform Ru (0001), which is the reference electrocatalyst, and to be selective to NRR as it shows very poor activity toward the HER reaction. It is worth pointing out that the better performance of this system normally is due to the participation of hydrogen atoms of −OH groups, stabilizing reaction intermediates, and thereby helping at reducing the energetic costs of the reaction. Note, in addition, that according to computationally derived Pourbaix diagrams, such situations are predicted to be stable under working conditions.

## Supporting Information Available

Surface Pourbaix diagrams me thod; thermodynamic cycle for evaluating the stability of B atom; stability-related energies and Bader charge of different moieties; N_2_ adsorption models, energies and bond lengths, and Bader charge changes between after N_2_ adsorption; limiting potential (*U*_L_) and steps for the studied models; charge density difference plots of inspected models; geometric structures of N_2_ adsorption; and total and partial density of states of inspected models considering terminations; Pourbaix diagrams for models considering both terminations and B atom (PDF)
