# Supplementary material for: Unveiling the Synergy between Surface Terminations and Boron Configuration in Boron-Based Ti3C2 MXenes Electrocatalysts for Nitrogen Reduction Reaction
Source: ACS Catal. 2024 Oct 3;14(20):15429–43. doi: 10.1021/acscatal.4c03415 (PMC11494508; doi:10.1021/acscatal.4c03415)
Supplement: Supplementary file 1 — cs4c03415_si_001.pdf [file cs4c03415_si_001.pdf]

Unveiling the Synergy between Surface Terminations and Boron Configuration in Boron-Based Ti_3C_2 MXenes Electrocatalysts for Nitrogen Reduction Reaction

Ling Meng, Francesc Viñes, and Francesc Illas*

Departament de Ciència de Materials i Química Física & Institut de Química Teòrica i Computacional (IQTCUB), Universitat de Barcelona, c/ Martí i Franquès 1-11, 08028, Barcelona, Spain

*corresponding author: francesc.illas@ub.edu

Section S1: Surface Pourbaix diagrams

The purpose of constructing the Pourbaix diagram in this study is to identify the most thermodynamically stable surface composition of Ti_3C_2 MXene (0001) during NRR under realistic working conditions (*pH* and *U*). Note that various distributions of terminations and boron-doped atom were considered, taking also into account the number of electrons (e^-) and protons (H^+) involved, the stoichiometric coefficients $v(e^-)$ and $v(H^+)$ were incorporated into the reaction formation free energy equations required for constructing the Pourbaix diagram, as

$$\Delta G(pH,U) = \Delta G(0,0) - \upsilon(H^+) k_B T \cdot \ln 10 \cdot pH - \upsilon(e^-) eU \quad (1),$$

where $\Delta G(0,0)$ is the formation energy of a given surface termination at zero *pH* and *U*, *U* refers to the applied electrode potential relative to the reversible hydrogen electrode (RHE), *e* is the electron charge, and k_B is Boltzmann's constant. To account for the chemical potential of electron-proton pairs, we rely on the computational hydrogen electrode (CHE) model; however, for B atom, we use the standard electrode potential as,

$$B(OH)_3 + 3H^+ + 3e^- \rightleftharpoons B_{(s)} + 3H_2O \quad (2),$$

where, according to $\Delta G = -nF\Delta E°$ where $\Delta E° = -0.89$ V and the boron bulk $B_{(s)}$ energy, the energy of ($B^{3+} + 3e^-$) can be obtained, then incorporate this energy into the formation Gibbs free energy. Using this method, a Pourbaix diagram can be plotted, showing which surface composition, has the lowest ΔG value under any *pH* and *U* conditions. We also refer the reader to recent publications by the authors.[1,2] The surface composition with the lowest ΔG is illustrated in Figure S10.

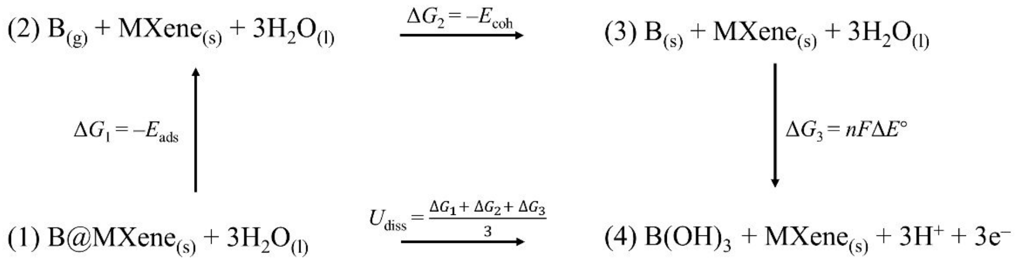

Scheme S1. Thermodynamic cycle to determine the Gibbs free energy of the Boron active site dissolution process.

Table S1. Calculated formation energies per atom, E_f, B adsorption energies, E_{ads}, and the E_{diff} value, all given in eV. The Bader charges of the MXene, Q_{MXene}, the doping B atom, Q_B, and the terminating groups, Q_{T_x}, is given, in *e*.

Model	E_f	E_{ads}	E_{diff}	U_{diss}	Q_{MXene}	Q_{T_x}	Q_B
**s**B-O	-0.39	-2.53	3.93	-2.20	1.03	-0.98	-0.06
**s**B-OH	-0.37	-4.30	2.15	-1.61	0.74	-0.62	-0.12
**a**B-O	-0.54	-7.66	-1.20	-0.49	0.96	-1.20	0.24
**a**B-H_C-O_4	-0.51	-6.09	0.36	-1.01	0.75	-0.99	0.24
**a**B-B_O-O_4	-0.49	-4.73	1.73	-1.47	0.78	-0.96	0.18
**a**B-H_C-O_3	-0.50	-5.77	0.68	-1.12	0.70	-0.94	0.24
**a**B-B_O-O_3	-0.48	-4.47	1.99	-1.55	0.75	-0.92	0.17
**a**B-O_2	-0.46	-4.04	2.41	-1.69	0.71	-0.87	0.16
**a**2B-O	-0.51	-6.42	0.04	-0.90	0.90	-1.23	0.16
**a**2B-OH	-0.47	-5.39	1.07	-1.25	0.71	-1.00	0.15

Table S2. Estimated N_2 adsorption energies, E_{ads}, on the studied models, including physisorption, -*p*, and chemisorption states, including *end-on*, -*e*, *side-on*, -*s*, *bridge-end-on*, -*be*, and *bridge-side-on*, -*bs* bonding modes. Bader charges on N_2 adsorbed species, Q_{N_2}, and change in doping B atom Bader charge, ΔQ_B, both in *e*, and smallest bond length between the surface B and the adsorbed N_2 molecule, *d*(BN), and of the N_2 molecule, *d*(NN), both in Å. The computed $N_2^{(g)}$ bond length is 1.12 Å for reference.

**Symbols**	E_{ads}	ΔQ_B	Q_{N_2}	***d*(BN)**	***d*(NN)**
**s**B-O-*e*	-1.87	0.08	-0.08	1.38	1.16
**s**B-O-*s*	-1.43	0.12	-0.12	1.48	1.26
**s**B-OH-*e*	-2.79	0.12	-0.15	1.35	1.21
**s**B-OH-*s*	-1.52	0.18	-0.20	1.47	1.40
**a**B-O-*p*	-0.09	0.00	0.00	3.26	1.12
**a**B-H_C-O_4-*p*	-0.12	0.00	0.00	3.30	1.12
**a**B-B_O-O_4-*e*	-0.68	0.06	-0.07	1.46	1.16
**a**B-B_O-O_4-*s*	-0.37	0.05	-0.12	1.56	1.24
**a**B-H_C-O_3-*p*	-0.12	0.00	0.00	3.26	1.12
**a**B-B_O-O_3-*e*	-0.67	0.07	-0.08	1.44	1.17
**a**B-O_2-*e*	-0.78	0.07	-0.10	1.42	1.18
**a**B-O_2-*s*	-1.19	0.07	-0.15	1.52	1.27
**a**2B-O-*e*	-0.07	0.01	-0.02	1.60	1.12
**a**2B-O-*be*	0.30	0.06	-0.12	1.62	1.21
**a**2B-O-*bs*	-0.71	0.09	-0.14	1.52	1.25
**a**2B-OH-*p*	-0.30	0.00	0.00	3.51	1.12
**a**2B-OH-*bs*	-0.55	0.09	-0.18	1.49	1.29

Table S3. Calculated limiting potential, U_L, corresponding to the PDS in NRR, its PDS electrochemical step, and the U_L of the HER.

**Symbols**	**PDS**	**U_L(NRR)**	**U_L(HER)**
**s**B-O	$NH_2^* \rightarrow NH_3^*$	-1.40	-1.28
**s**B-OH	$NH_2^* \rightarrow NH_3^*$	-1.47	-1.19
**a**B-O	$N_2^* \rightarrow N_2H^*$	-1.37	-0.31
**a**B-O_4	$NH_2^* \rightarrow NH_3^*$	-1.19	-1.61
**a**B-O_3	–O* → –OH*	-0.83	-1.75
**a**B-O_2	–O* → –OH*	-2.01	-1.67
**a**2B-O	$NH_2^* \rightarrow NH_3^*$	-1.51	-0.10
**a**2B-OH	$NH_2^* \rightarrow NH_3^*$	-2.10	-0.46

Figure S1. Top view contour maps of charge density difference (CDD) plots for the studied models derived from Figure 1 of main text, along the T_x plane. Yellowish regions denote electron depletion, *i.e.* the formation of positively charged regions, while blueish regions denote electron accumulation, and the formation of negatively charged regions. The contour intervals range up to 0.01 $e{\cdot}Å^{-3}$.

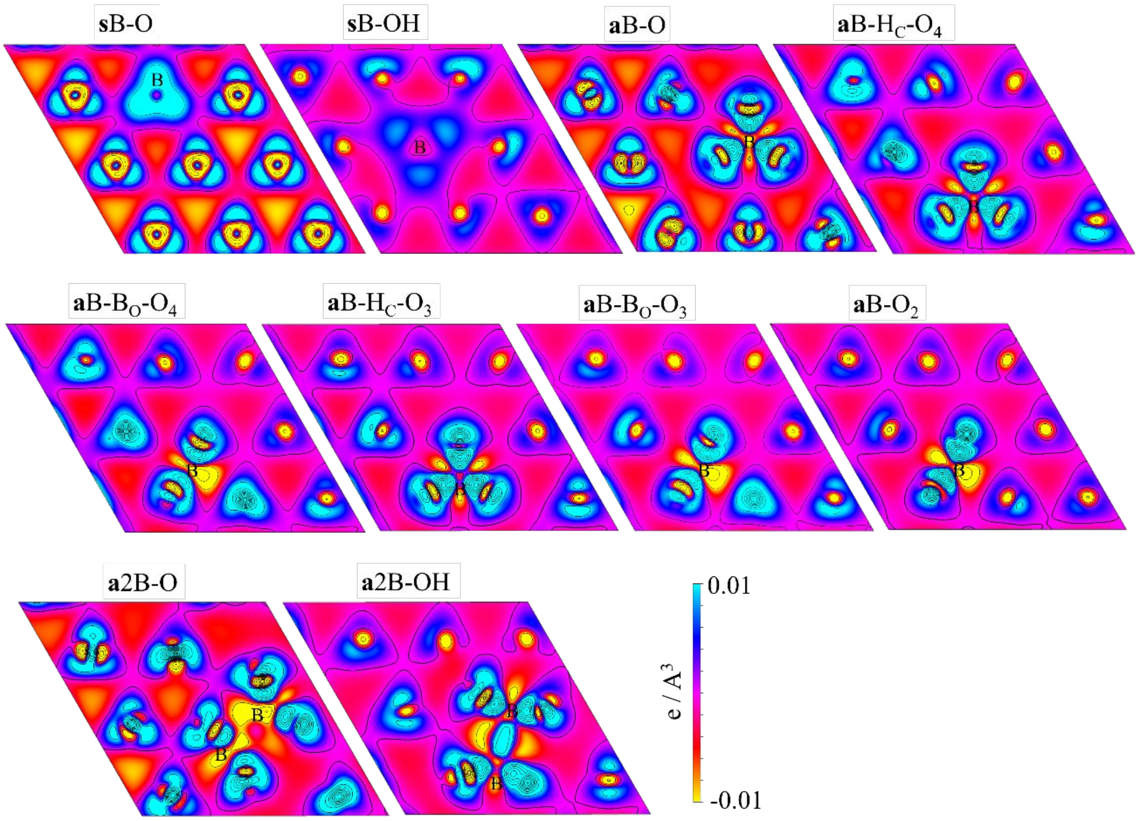

Figure S2. Total DOS and projected DOS (PDOS) of the studied models. Energy levels are referred to the Fermi energy, E_F, set to zero.

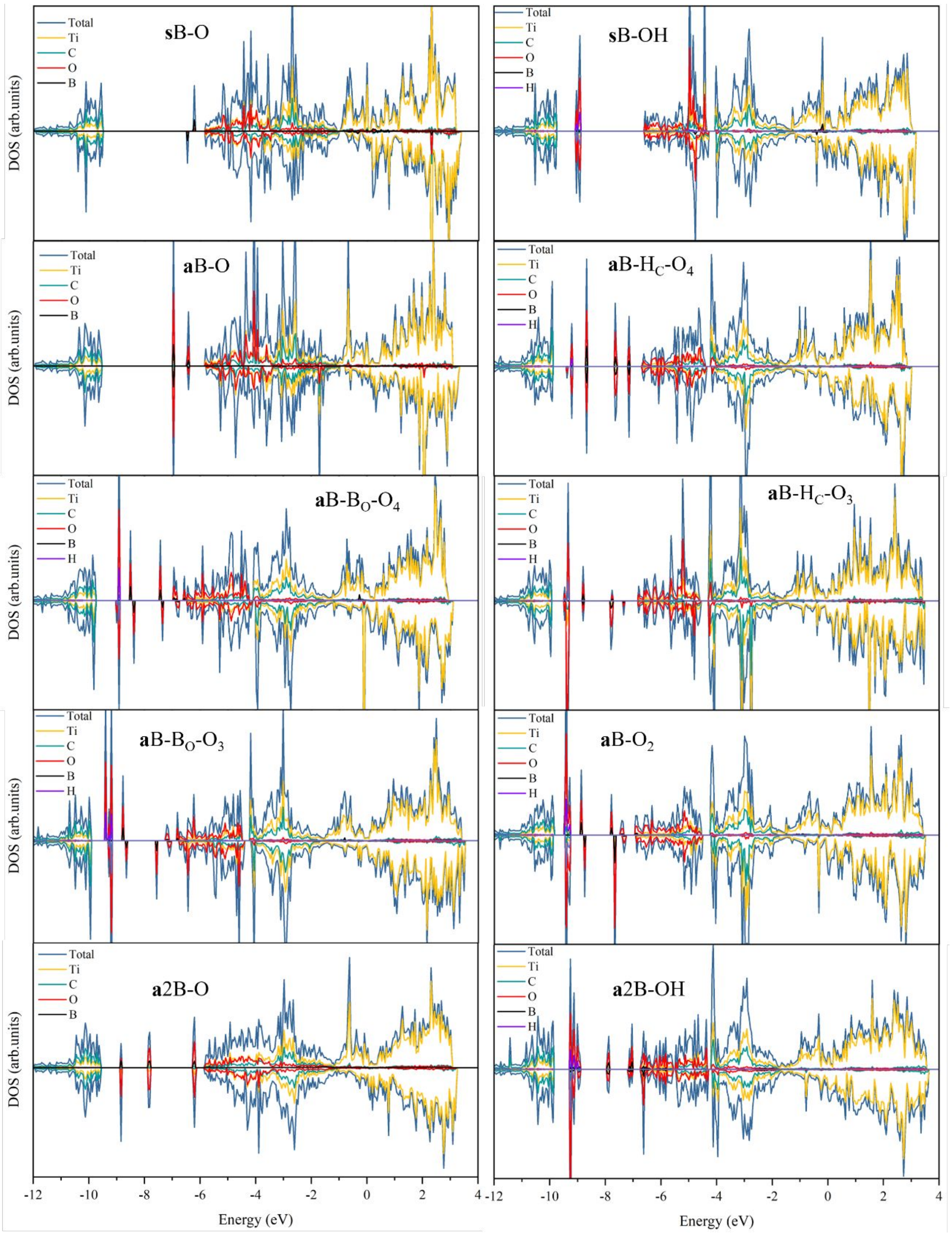

Figure S3. Optimized structures of N_2 adsorbed on the studied models shown in Figure 1 of the main text. N_2 adsorption modes are tagged in italics: -*e* (*end-on*), -*s* (*side-on*), -*be* (*bridge-end-on*), and -*bs* (*bridge-side-on*) for chemisorption, and -*p* for physisorption. Elements are color-coded as in Figure 1 of the main text, while N atoms are shown in light blue color.

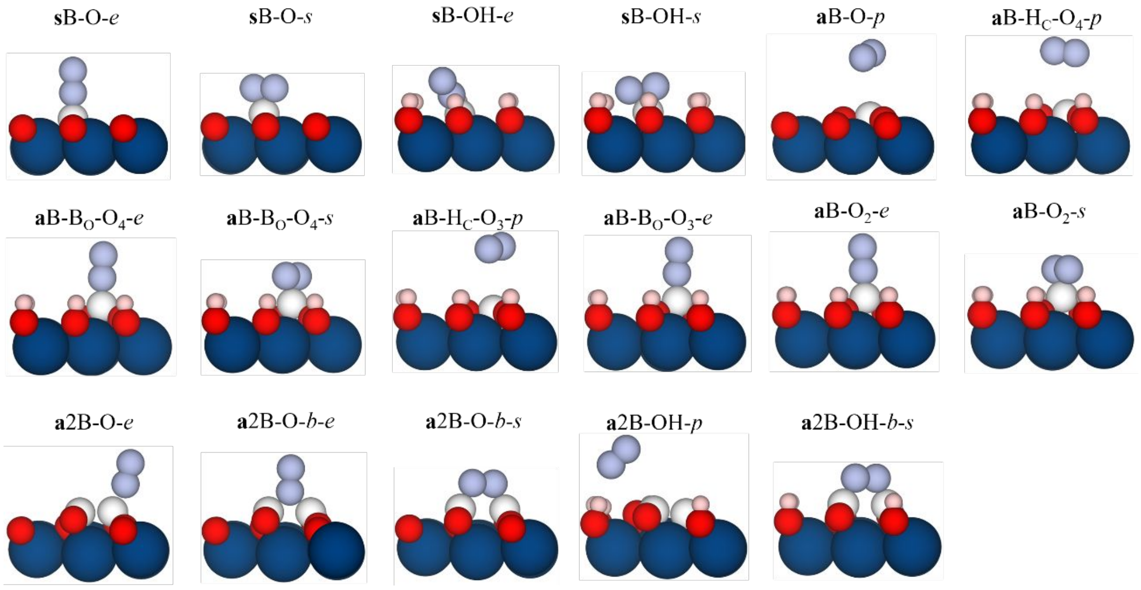

Figure S4. Projected density of states (PDOS) of the studied substituted boron (**s**B) models shown in Fig. S3, distinguishing between B and N contributions. Energy levels are referred to Fermi energy, E_F, set to zero.

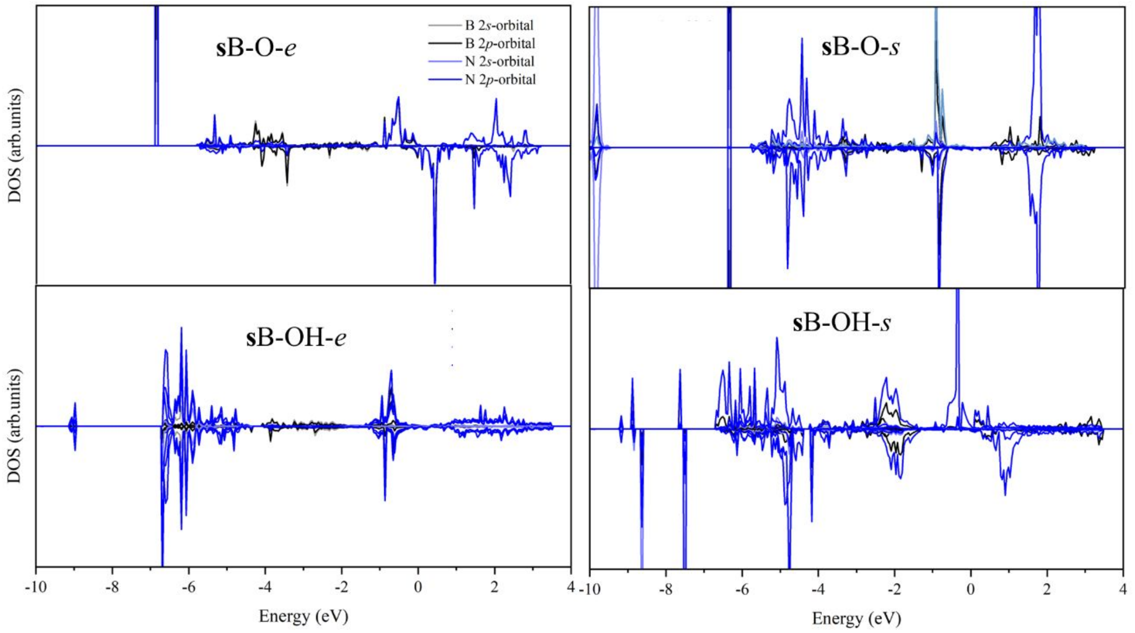

Figure S5. PDOS of the studied adsorbed boron (**a**B) models shown in Fig. S3, distinguishing between B and N contributions. Energy levels are referred to Fermi energy, E_F, set to zero.

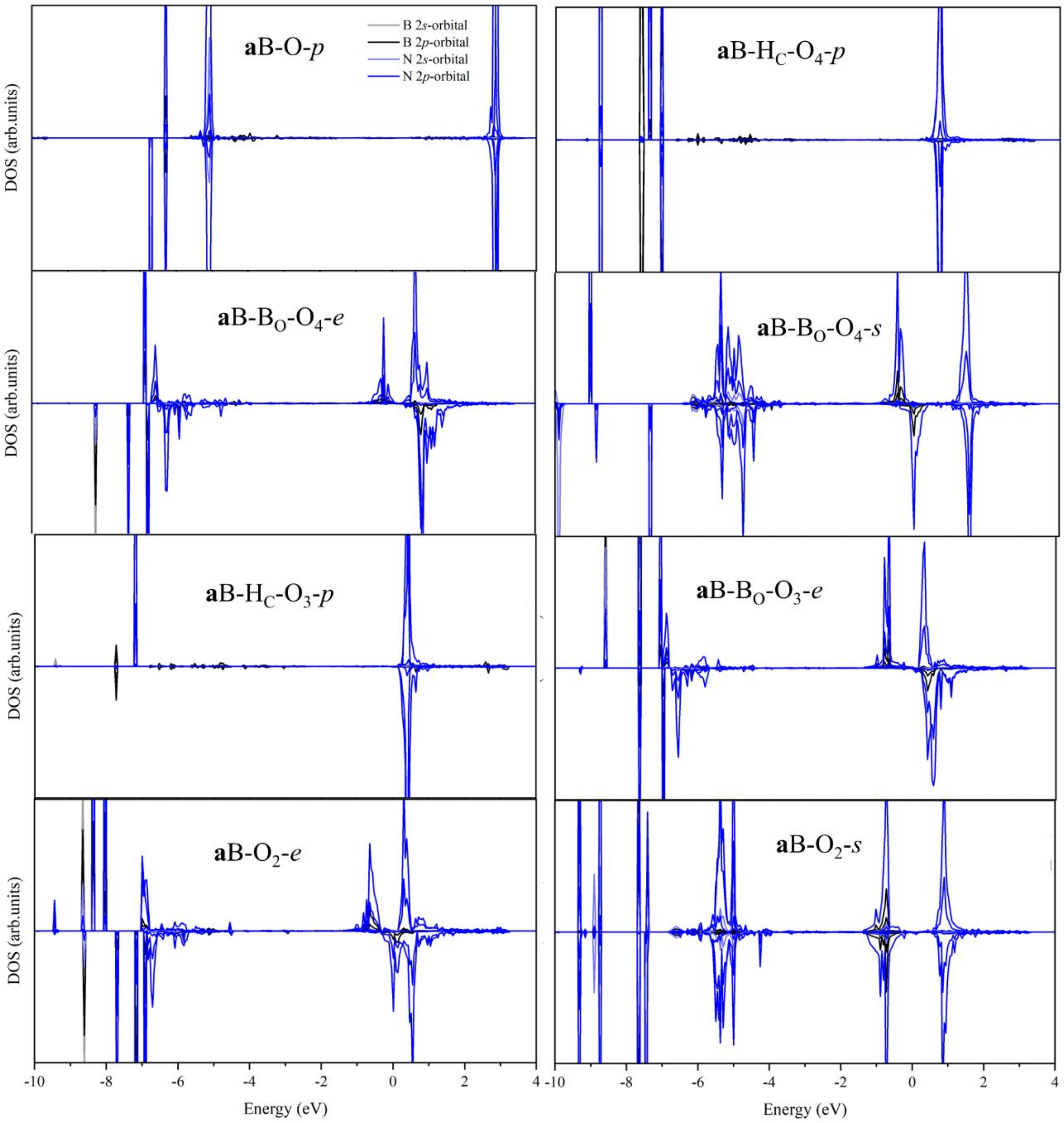

Figure S6. PDOS of the adsorbed boron dimers (**a**2B) models shown in Fig. S3, distinguishing between B and N contributions. Energy levels are referred to Fermi energy, E_F, set to zero.

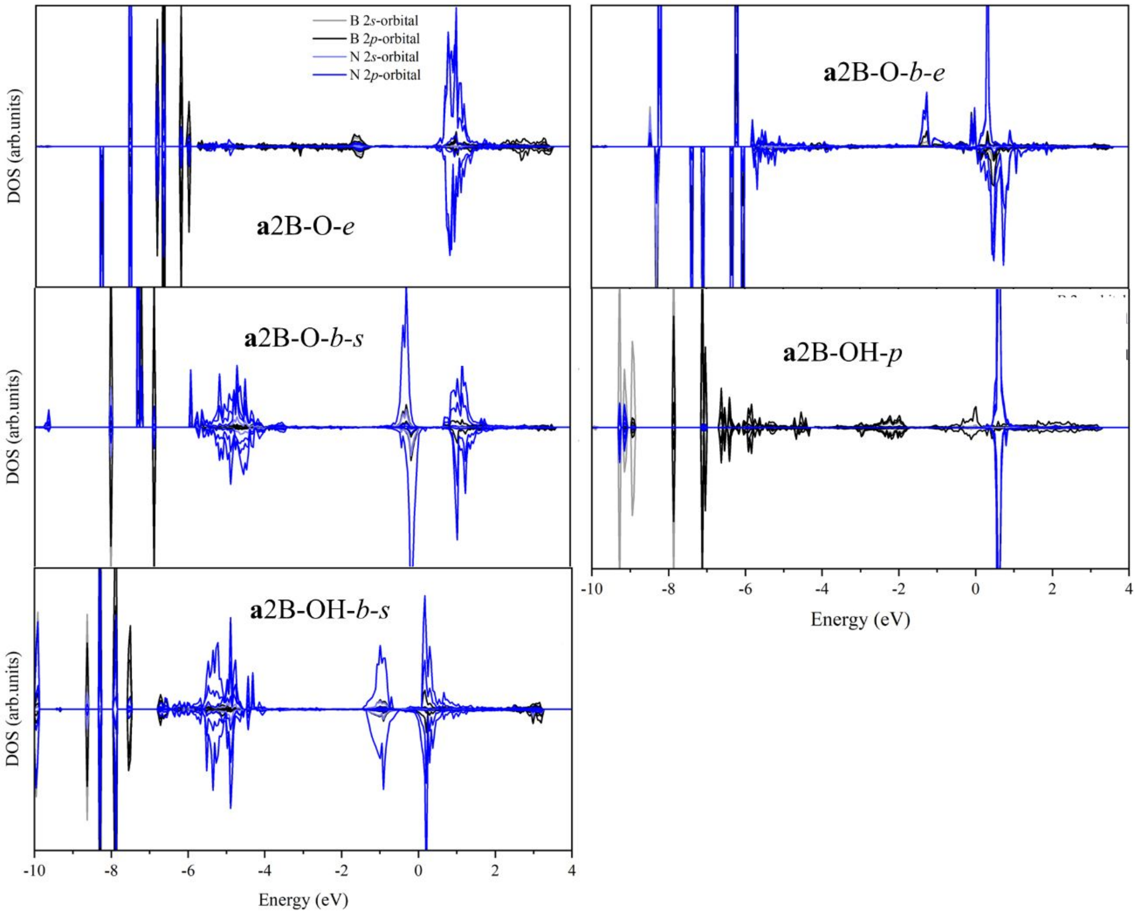

Figure S7. Side views of charge density difference (CDD) plots of the studied models shown in Fig. S3. Yellow and blue isocontours denote electron density depletion and accumulation, respectively. The contours are taken at isovalues of 0.001 $e{\cdot}Å^{-3}$. Elements are color-coded as in Figure S3.

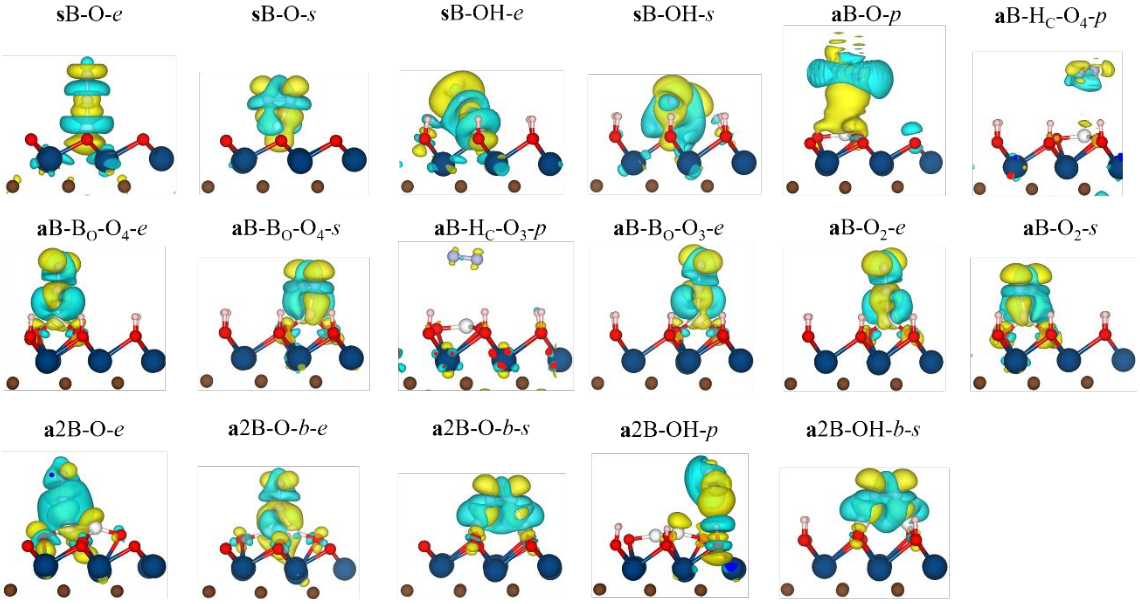

Figure S8. The alternative Gibbs free energy, ΔG, diagrams of NRR on **a**2B-OH under standard working conditions of T = 300 K, partial gases pressures of 1 bar, pH = 0, and U = 0 V. Solid lines represent chemical steps such as $N_2^{(g)}$ adsorption or as-generated $NH_3^{(g)}$ desorption, while dashed lines represent CPET steps. T_N represents adsorption on top of a nitrogen atom and T_B represents adsorption on a bridge between two nitrogen atoms. Below the reaction paths, side views of the atomic models for the different reaction steps are shown. Colour coding is as in Figure 5.

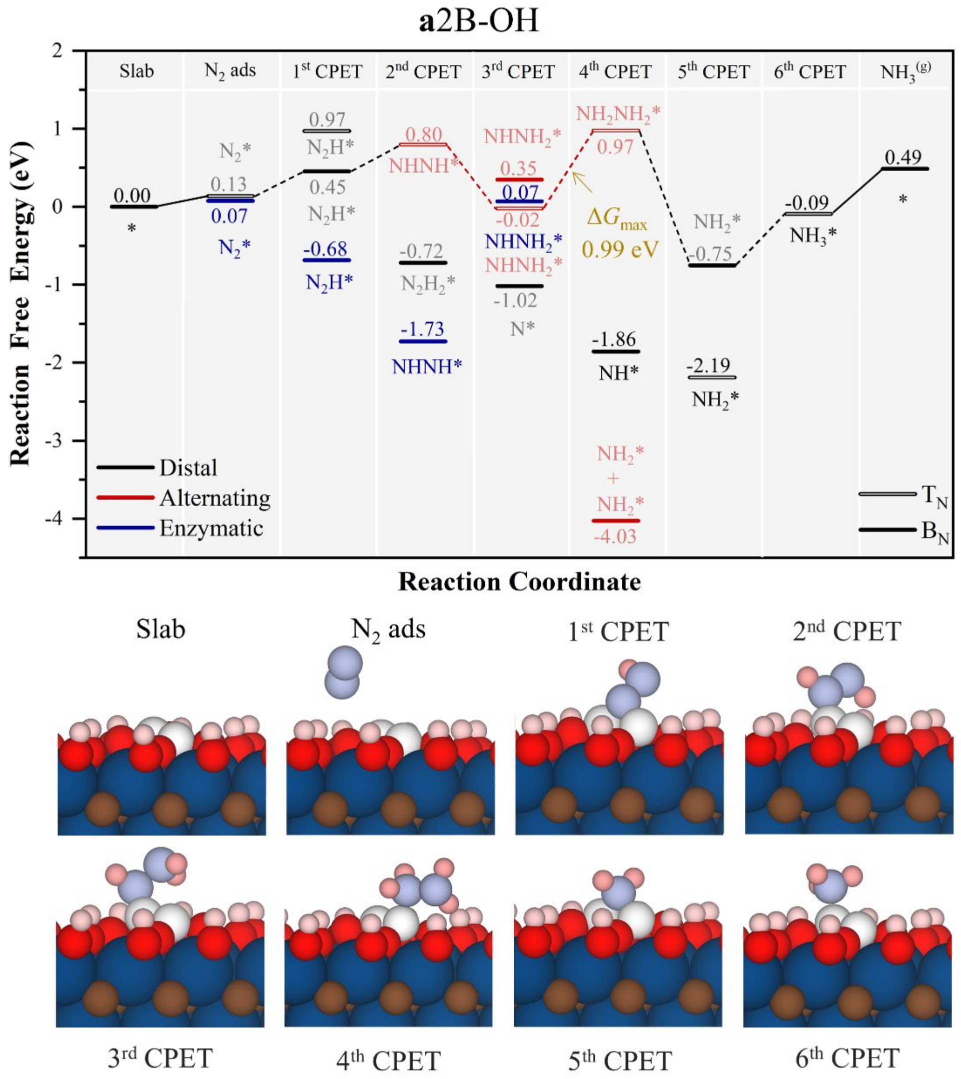

Figure S9. Pourbaix diagrams for the Ti_3C_2 MXene (0001) surface regarding single, binary surface compositions including –O, –OH, terminations. The black, dashed line indicates the HER equilibrium potential with respect to the RHE reference.

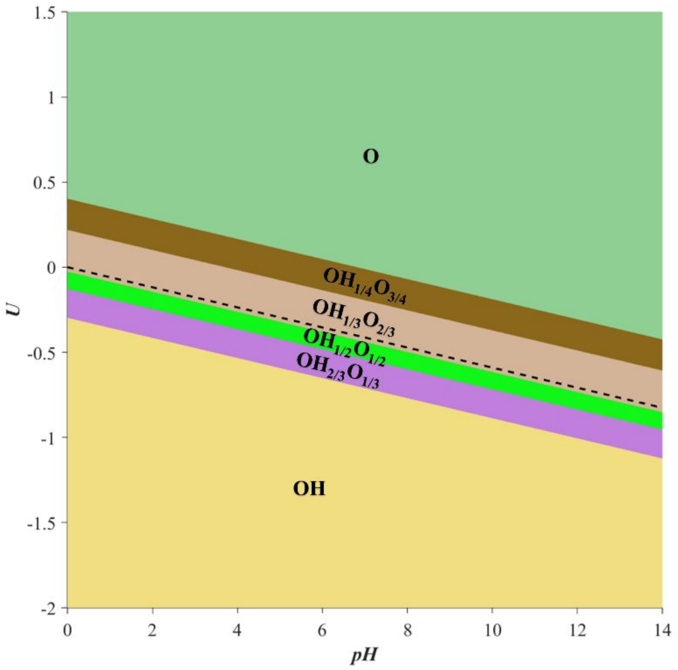

Figure S10. Pourbaix diagrams for the Ti_3C_2 MXene (0001) surface regarding –O, –OH, terminations and doped-B atom. The black, dashed line indicates the HER equilibrium potential with respect to the RHE reference.

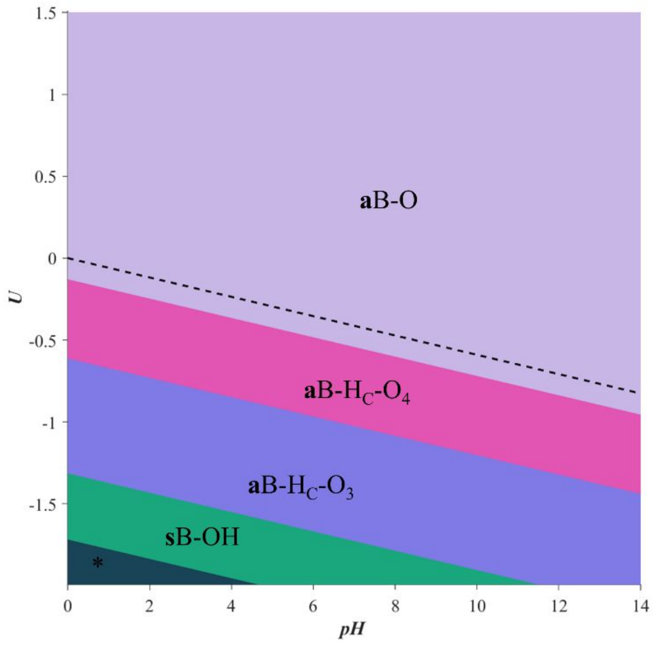

References

(1) Meng, L.; Yan, L.-K.; Viñes, F.; Illas, F. Effect of Terminations on the Hydrogen Evolution Reaction Mechanism on Ti3C2 MXene. *J. Mater. Chem. A* **2023,** *11*, 6886−6900.

(2) Meng, L.; Yan, L.-K.; Viñes, F.; Illas, F., Surface Termination Dependent Carbon Dioxide Reduction Reaction on Ti_3C_2 MXene. *J. Mater. Chem. A* **2024**, *12*, 7856−7874.
